# MEK1/2 inhibition transiently alters the tumor immune microenvironment to enhance immunotherapy efficacy against head and neck cancer

**DOI:** 10.1136/jitc-2021-003917

**Published:** 2022-03-15

**Authors:** Manu Prasad, Jonathan Zorea, Sankar Jagadeeshan, Avital B Shnerb, Sooraj Mathukkada, Jebrane Bouaoud, Lucas Michon, Ofra Novoplansky, Mai Badarni, Limor Cohen, Ksenia M Yegodayev, Sapir Tzadok, Barak Rotblat, Libor Brezina, Andreas Mock, Andy Karabajakian, Jérôme Fayette, Idan Cohen, Tomer Cooks, Irit Allon, Orr Dimitstein, Benzion Joshua, Dexin Kong, Elena Voronov, Maurizio Scaltriti, Yaron Carmi, Cristina Conde-Lopez, Jochen Hess, Ina Kurth, Luc G T Morris, Pierre Saintigny, Moshe Elkabets

**Affiliations:** 1 The Shraga Segal Department of Microbiology, Immunology, and Genetics, Ben-Gurion University of the Negev, Beer-Sheva, Israel; 2 Faculty of Health Sciences, Ben-Gurion University of the Negev, Beer-Sheva, Israel; 3 Department of Translational Medicine Oncology, Centre Léon Bérard, Lyon 69373, France; 4 Univ Lyon, Université Claude Bernard Lyon, INSERM 1052, CNRS 5286, Centre Léon Bérard, Centre de Recherche en Cancérologie de Lyon, Lyon 69373, France; 5 Department of Life Sciences, Ben-Gurion University of the Negev, Beer-Sheva, Israel; 6 Department of Medical Oncology, Heidelberg University Hospital, Heidelberg, Germany; 7 Division of Translational Medical Oncology, NCT Heidelberg, German Cancer Research Centre (DKFZ), Heidelberg, Germany; 8 Department of Medical Oncology, Centre Léon Bérard, Lyon 69373, France; 9 Institute of Pathology, Barzilai University Medical Center, Ashkelon, Israel; 10 Department of Otolaryngology-Head & Neck Surgery, Soroka University Medical Center, Beer-Sheva, Israel; 11 Department of Otorhinolaryngology and Head & Neck Surgery, Barzilai Medical Center, Ashkelon, Israel; 12 School of Pharmaceutical Sciences, Tianjin Medical University, Tianjin, China; 13 Human Oncology and Pathogenesis Program, Memorial Sloan Kettering Cancer Center, New York City, New York, USA; 14 Department of Pathology, Tel Aviv University, Tel Aviv, Israel; 15 Division of Radiooncology-Radiobiology, German Cancer Research Center (DKFZ), Heidelberg, Germany; 16 Section Experimental and Translational Head and Neck Oncology, Department of Otolaryngology, Head and Neck Surgery, University Hospital Heidelberg, Heidelberg, Germany; 17 Research Group Molecular Mechanisms of Head and Neck Tumors, German Cancer Research Center (DKFZ), Heidelberg, Germany; 18 Department of Surgery, Memorial Sloan Kettering Cancer Center, New York, New York, USA

**Keywords:** Head and neck cancer, tumor-microenvironment, tumor-immunity, immunotherapy, targeted therapy, MEK1/2, anti-PD-1

## Abstract

**Background:**

Although the mitogen-activated protein kinases (MAPK) pathway is hyperactive in head and neck cancer (HNC), inhibition of MEK1/2 in HNC patients has not shown clinically meaningful activity. Therefore, we aimed to characterize the effect of MEK1/2 inhibition on the tumor microenvironment (TME) of MAPK-driven HNC, elucidate tumor-host interaction mechanisms facilitating immune escape on treatment, and apply rationale-based therapy combination immunotherapy and MEK1/2 inhibitor to induce tumor clearance.

**Methods:**

Mouse syngeneic tumors and xenografts experiments were used to analyze tumor growth in vivo. Single-cell cytometry by time of flight, flow cytometry, and tissue stainings were used to profile the TME in response to trametinib (MEK1/2 inhibitor). Co-culture of myeloid-derived suppressor cells (MDSC) with CD8^+^ T cells was used to measure immune suppression. Overexpression of colony-stimulating factor-1 (CSF-1) in tumor cells was used to show the effect of tumor-derived CSF-1 on sensitivity to trametinib and anti-programmed death- 1 (αPD-1) in mice. In HNC patients, the ratio between CSF-1 and CD8A was measured to test the association with clinical benefit to αPD-1 and αPD-L1 treatment.

**Results:**

Using preclinical HNC models, we demonstrated that treatment with trametinib delays HNC initiation and progression by reducing tumor cell proliferation and enhancing the antitumor immunity of CD8^+^ T cells. Activation of CD8^+^ T cells by supplementation with αPD-1 antibody eliminated tumors and induced an immune memory in the cured mice. Mechanistically, an early response to trametinib treatment sensitized tumors to αPD-1-supplementation by attenuating the expression of tumor-derived CSF-1, which reduced the abundance of two CSF-1R^+^CD11c^+^ MDSC populations in the TME. In contrast, prolonged treatment with trametinib abolished the antitumor activity of αPD-1, because tumor cells undergoing the epithelial to mesenchymal transition in response to trametinib restored CSF-1 expression and recreated an immune-suppressive TME.

**Conclusion:**

Our findings provide the rationale for testing the trametinib/αPD-1 combination in HNC and highlight the importance of sensitizing tumors to αPD-1 by using MEK1/2 to interfere with the tumor–host interaction. Moreover, we describe the concept that treatment of cancer with a targeted therapy transiently induces an immune-active microenvironment, and supplementation of immunotherapy during this time further activates the antitumor machinery to cause tumor elimination.

## Introduction

Genomic alterations in genes of the mitogen-activated protein kinase (MAPK) pathway, or activation of receptor tyrosine kinases (RTK), induce constant activation of MEK-ERK signaling, which regulates cell proliferation, survival, migration, and transformation.^1 2^ Since hyperactivation of the MAPK pathway is involved in the pathogenesis of head and neck cancer (HNC)^3 4^ ^5^, blockade of the MAPK pathway has been studied as a therapeutic approach for counteracting HNC progression. For example, cetuximab, an antiepidermal growth factor receptor (EGFR) antibody that blocks the MAPK pathway, showed a significant but transient antitumor effect in HNC patients.^6^ Currently, multiple clinical trials are testing MAPK/RTK inhibitors in combination with different modalities in patients with HNC. Trametinib, a MEK1/2 kinase inhibitor approved for treatment of MAPK-driven melanoma and non-small cell lung cancer, exhibited a favorable but transient antitumor effect in HNC patients with oral squamous cell carcinoma (SCC).^7^ Trametinib, and other blockers of the MAPK pathway, act by arresting the growth of MAPK-driven tumor cells, but they also affect the host immunity and the heterogeneity of the tumor microenvironment (TME).^8–10^ The TME is composed of different stromal and immune cell types, which interact with each other and with the malignant cells and thus determine tumor progression (reviewed in^11 12^). The complex communication network between malignant cells and the TME is mediated, in part, by the tumor cell-derived cytokines and chemokines that regulate the heterogeneity of the TME and support tumor progression and/or resistance to therapy (13 and reviewed in^14–16^). In preclinical models, MEK inhibition was shown to delay the progression of MOC1 and MOC2 tumors in mice,^17 18^ and in SCC-VII HNC model,^19^ it was shown that treatment of tumor-bearing mice with trametinib induced CD8^+^ T cell infiltration into the TME and upregulated expression of programmed death-ligand 1 (PD-L1) by the tumor cells. It was also shown that combined treatment with trametinib and anti-PD-L1 antibody (αPD-L1) arrested tumor growth in the mice.^19^ While the above lines of evidence support the involvement of MAPK inhibitors in regulating the heterogeneity of TME and thus in determining antitumor immunity, the mechanisms of the communication network between the malignant cells and the TME are yet to be fully understood.

Here, we found that trametinib treatment delays tumor initiation and progression of MAPK-pathway mutated HNC, while altering the heterogeneity of TME, in part via downregulating the expression of tumor-derived colony-stimulating factor-1 (CSF-1). Tumor-derived CSF-1 controlled the quantity of CSF-1R^+^CD11c^+^ myeloid-derived suppressor cells (MDSCs) and the infiltration of activated CD8^+^ T cells into the tumor, which subsequently affected the sensitivity to the FDA-approved immunotherapy for HNC, anti-PD-1 (αPD-1).^20^ We showed that the timing of αPD-1 supplementation to trametinib treatment is crucial, as tumor elimination occurred only when αPD-1 supplementation was administered in the time window during which trametinib treatment had temporarily reduced CSF-1 expression and induced an immune active TME. This transient immune activation, reflected by low CSF-1 expression and high CD8A expression, was positively associated with a clinical benefit for HNC patients treated with αPD-1.

### Materials and methods

Provided as a online supplemental file 1

10.1136/jitc-2021-003917.supp1 Supplementary data

## Results

### MAPK pathway blockade with trametinib delays HNC initiation and progression

We first established the frequency of genomic alterations in the genes associated with the MAPK pathway in multiple HNC cohorts by interrogating available databases and found that between 5% and 50% of genes are altered (online supplemental figure S1A and online supplemental table S1). Targeted sequencing of 1680 HNC patients (AACR GENIE cohort), and whole-exome sequencing of 676 HNC patients showed that the frequency of mutations in MAPK related genes was 13% and 29%, respectively (online supplemental figure S1B, C). Moreover, inference of pathway activity based on RNA sequencing (RNA-seq) data from The Cancer Genome Atlas (TCGA)-HNC revealed significant associations of MAPK pathway hyperactive HNC with a worse prognosis, a larger tumor size, a basal classified signature, and human papillomavirus 16-negative (HPV16^-^) status as compared with patients with low MAPK pathway activity (online supplemental figure S1D and online supplemental tables S2 and S3). In addition, the expression of ERK2, a key component of the MAPK signaling pathway that is encoded by the *MAPK1* gene, is upregulated in HNC tumors compared with normal tissue, and there is an association between the extent of upregulation and the tumor grade (online supplemental figure S1E, F) (UALCAN^21^-TCGA data). Lastly, analysis of the cancer cell line encyclopedia and the Sanger cell line datasets showed that HNC cell lines are sensitive to trametinib (online supplemental figure S1G), a conclusion supported by the recent publication of Lepikhova *et al.*^22^

10.1136/jitc-2021-003917.supp2 Supplementary data

10.1136/jitc-2021-003917.supp3 Supplementary data

**Figure 1:**
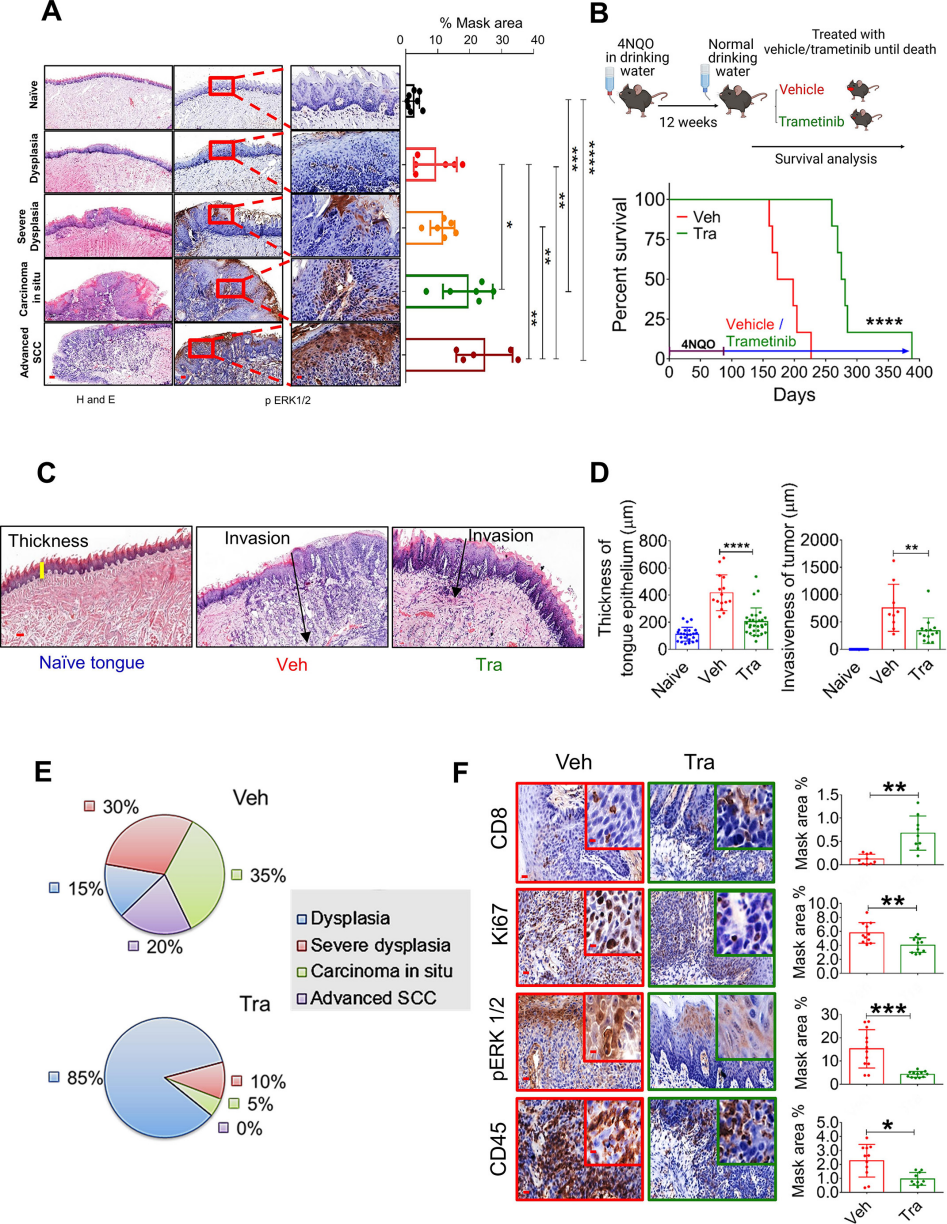
MAPK pathway is hyperactive in HNC, and trametinib induced MAPK pathway blockage delays HNC initiation and progression. (A) H&E (left) and pERK1/2 (right) staining at various stages of oral (tongue) carcinogenesis induced by 4NQO in C57BL/6 J mice. Percentage mask area is shown on the right. Scale bars: 200 µm (left); 100 µm (middle); and 20 µm (right). (B) Top—scheme of the experimental setting investigating the survival of 4NQO-treated mice subsequently treated with trametinib or vehicle. Bottom—survival rates of immunocompetent C57BL/6 J mice (n=6) in a 4NQO cancer model following daily treatments with vehicle or trametinib (1 mg/kg/day). (C) H&E images and (D) statistics for the tongues showing the thickness of the margins and invasion of the tumors (scale bars: 100 µm). (E) Pie diagrams showing the percentages of different cancer grades in vehicle- and trametinib-treated mice. (F) IHC images showing the infiltration of CD45^+^ cells, CD8^+^ T cells, and the expression of pERK1/2 and Ki67 in the tongues of 4NQO exposed mice treated with vehicle or trametinib (scale bars: 20 µm; inset 10 µm). For statistics, an unpaired two-sided t-test or one-way ANOVA was performed. *P<0.05; **p<0.01; ***p<0.001, ****p<0.0001 were considered statistically significant. ANOVA, analysis of variance; HNC, head and neck cancer; IHC, immunohistochemistry; MAPK, mitogen-activated protein kinase; Tra, trametinib; Veh, vehicle.

Following the above-described analysis of the published data, we assessed the efficacy of trametinib in two independent HNC patient-derived xenografts (PDXs) in which trametinib treatment either stabilized or significantly delayed tumor growth (online supplemental figure S1H). Immunohistochemistry (IHC) analysis of the PDXs showed that trametinib reduced cell proliferation (Ki67 staining) and phosphorylated ERK1 and 2 (pERK1/2) levels as a 'readout' of MAPK pathway activation (online supplemental figure S1I). To investigate the impact of MAPK pathway activation on HNC initiation and progression in preclinical models, we initially determined pERK1/2 levels at various stages of oral carcinogenesis induced by the mutagenic compound 4-nitroquinoline-1-oxide (4NQO)^23^ in C57BL/6J wild-type (WT) mice. Histopathological analysis of the tongues of 4NQO-treated mice showed a significant increase in pERK1/2 levels from lower pathological grades^11^ (dysplasia) to higher pathological stages, namely, advanced SCC (figure 1A). Higher pERK1/2 levels and more advanced pathological grades were associated with increased cell proliferation, as indicated by Ki67 staining (online supplemental figure S1J). To explore whether targeting the MAPK pathway could be used for chemoprevention, we exposed WT mice to 4NQO for 12 weeks and then treated them with either trametinib or vehicle (figure 1B). The vehicle-treated mice started to develop physical signs of tumor progression, manifested by weight loss and swallowing difficulties after 14 weeks (~100 days) and began to die after 160 days (figure 1B). In contrast, trametinib-treated mice started to show signs of disease onset after as long as 250 days. All vehicle-treated mice died within 220 days, while trametinib-treated mice survived significantly longer, with an extension of 100 days in median survival (figure 1B). An independent experiment in which we monitored disease progression and histopathology of mouse tongues on day 150 further confirmed that trametinib delayed tumor development (online supplemental figure S1K). In the vehicle-treated group, we detected 4–6 macroscopic lesions on the tongue of each mouse, whereas in the trametinib-treated group only 1–2 lesions per mouse were observed (online supplemental figure S1K). Moreover, mice treated with trametinib exhibited a less invasive phenotype, as determined by the depth of invasion and reduced hyperplasia of the basal layer of the tongue^24^ (figure 1C and D). Analysis of the tongue lesions showed the development of carcinoma in situ and advanced SCC in 55% of vehicle-treated mice, compared with only 5% of trametinib-treated mice (figure 1E).

Since previous studies have shown that trametinib treatment modulates the TME, thereby enhancing the antitumor effect of CD8^+^ T cells,^19 25–28^ we stained the vehicle-treated and trametinib-treated tongues with antibodies against the lymphocyte markers CD45 and CD8. IHC analysis of the tongues indicated that the vehicle-treated group was enriched with CD45^+^ cells but the number of CD8^+^ T cells was significantly lower than that in the trametinib-treated group (figure 1F). These results suggest that trametinib treatment delays tumor progression by inhibiting the MAPK pathway in the tongue epithelium and reducing cell proliferation. This delay in malignancy development is also associated with an enrichment of CD8^+^ T cells in the 4NQO-exposed epithelium.

### CD8^+^ T cells are required for a prolonged response to trametinib

**Figure 2:**
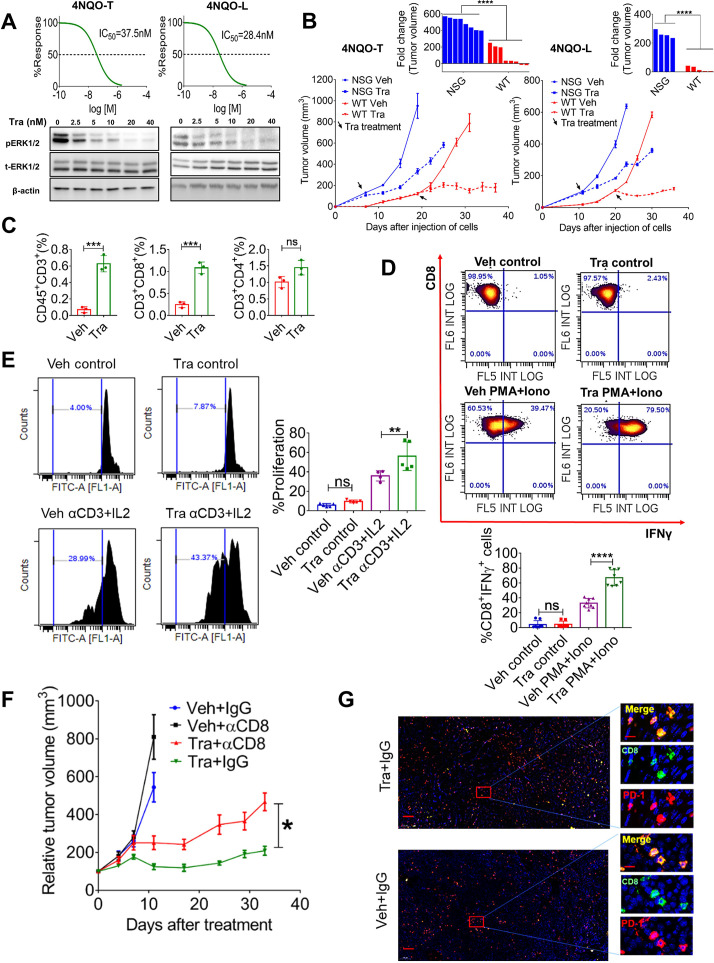
Trametinib treatment induces infiltration of activated CD8^+^ T cells leads to prolonged tumor growth arrest. (A) Top—viability of 4NQO-T and 4NQO-L cell lines treated with increasing doses of trametinib for 4 days; IC_50_ values are shown. Bottom—Western blot analysis showing the expression levels of pERK1/2, total ERK1/2 (t-ERK1/2), and beta-actin (as the loading control) after treatment with increasing doses of trametinib for 24 hours in 4NQO-T and 4NQO-L cell lines. (B) Growth curve and statistics of 4NQO-T and 4NQO-L tumors in NSG and WT mice treated with vehicle or trametinib. Top—Fold change of tumor volumes of 4NQO-L and 4NQO-T tumors treated with trametinib. (C) Flow cytometry analysis of the lymphocytic population in the 4NQO-L tumors treated with vehicle or trametinib for 5 days (D) intracellular staining of IFNγ in CD8^+^ T cells isolated from the vehicle-treated or trametinib SE-treated (5 days) mice. Density plots showing the percentage of CD8^+^IFNγ^+^ with or without activation with phorbol 12-myristate 13-acetate (PMA) and ionomycin (Iono). Statistics of 3 independent experiments are shown below. (E) In vitro CFSC proliferation assay (αCD3+IL2 stimulation) of CD8^+^ T cells isolated from tumors of mice treated for 5 days with either vehicle or trametinib. Statistics of two independent experiment is shown on the right. (F) Growth of 4NQO-L tumors in WT mice treated with trametinib, with and without depletion of CD8^+^ T cells (n=5–6). (G) Immunofluorescence co-staining of CD8^+^ (green) and PD-1 (red) and the merged images (yellow) (scale bars: 200 µm; inset 10 µm). One way ANOVA was performed *p<0.05; **p<0.01; ***p<0.001, ****p<0.0001 were considered statistically significant. ANOVA, analysis of variance; CFSC, carboxyfluorescein diacetyl succinimidyl ester; Tra, trametinib; Veh, vehicle.

To explore whether the efficacy of trametinib is affected by host immunity, we used HNC cell lines derived from 4NQO-induced tumors.^29^ First, we evaluated the susceptibility of two 4NQO-induced murine HNC cell lines, 4NQO-T (tongue) and 4NQO-L (lip), to trametinib. The half inhibitory concentration (IC_50_) of trametinib was found to be ~37.5 nM and ~28.4 nM for 4NQO-T and 4NQO-L, respectively (figure 2A). Western blot analysis showed a dose-dependent reduction of pERK1/2 levels in both cell lines following trametinib treatment (figure 2A). Genomic sequencing of 468 cancer-related genes using the MSK-IMPACT platform^30^ showed that these murine cell lines harbor many of the mutated genes found in HNC cancer patients (online supplemental figure S2A and online supplemental table S4). Among these mutated genes, *KRAS* was the only MAPK-pathway related gene that was altered in both cell lines. Specifically, the 4NQO-T cell line harbors a mutation at G12A, while the 4NQO-L line harbors a mutation at G12C; both mutations are present in HNC patients (online supplemental figure S2B). To investigate the contribution of the immune system in the response to trametinib, we injected 4NQO-L and 4NQO-T cells into immunocompromised NOD/SCID/IL2rγ^null^ (NSG) and immunocompetent WT mice and compared tumor growth between the mice treated with trametinib and those receiving vehicle alone. In the NSG mice, trametinib treatment slowed tumor progression, while in the WT mice it induced a stable disease with no significant change in the tumor volume over the first 20 days (figure 2B). We observed similar results using another MAPK-driven HNC cell line KRAS^mut^EpT/C9Ep (online supplemental figure S2C).^31^ IHC analysis of the 4NQO-L tumors at the end of the experiment showed that while pERK1/2 staining was equally reduced in trametinib-treated NSG and WT mice, the proliferation rate of tumor cells was reduced in trametinib-treated WT mice compared with NSG mice (online supplemental figure S2D). In vitro, we detected upregulation of MHC class-I expression on the surface of 4NQO cell lines treated with trametinib, which was further increased in the presence of interferon-gamma (IFNγ) (online supplemental figure S2E). These in vivo and in vitro results suggest that the host antitumor immunity may enhance trametinib efficacy, thereby preventing tumor progression.

We then explored whether trametinib affects the infiltration of lymphocytes into the tumors. Analysis of the samples after 5 days of treatment with trametinib revealed a significant increase of CD8^+^ T cells but not CD4^+^ T cells when compared with vehicle-treated tumors (figure 2C). Thereafter, we examined the activation status of CD8^+^ T cells in tumors before and after trametinib treatment by comparing their ability to produce IFNγ in response to activation with phorbol 12-myristate 13-acetate (PMA) and ionomycin in vitro.^32 33^ Specifically, flow cytometry analysis of PMA and ionomycin-stimulated lymphocytes obtained from tumors of mice treated with vehicle or trametinib for 5 days showed that CD8^+^ T cells derived from trametinib-treated tumors expressed high levels of IFNγ compared with CD8^+^ T cells obtained from vehicle-treated tumors (figure 2D and online supplemental figure S2F, G). Furthermore, analysis of CD8^+^ T cell proliferation following anti-CD3/IL2 treatment shows that CD8^+^ T cells obtained from tumors treated with trametinib exhibited a greater proliferation capability than CD8^+^ T cells obtained from vehicle-treated tumors (figure 2E). The enhanced activity of CD8^+^ T cells was also associated with a reduction in the expression of TIM3 (T-cell immunoglobulin domain and mucin domain 3) and PD-1, which are markers of dysfunction and exhaustion of CD8^+^ T cells (online supplemental figure S2H).^34–36^

The next step was to investigate the role of CD8^+^ T cells in modulating trametinib efficacy by depleting CD8^+^ T cells in 4NQO-L tumor-bearing mice. Specifically, a cohort of WT 4NQO-L tumor-bearing mice was divided into four treatment arms, that is, vehicle +IgG, trametinib +IgG, vehicle +anti-CD8, and trametinib +anti-CD8. In the mice treated with anti-CD8 (ie, depleted of CD8^+^ T cells), tumor progression was rapid, compared with the IgG control groups, and in those depleted of CD8^+^ T cells and treated with trametinib the antitumor activity of trametinib was attenuated, as evidenced by 4 out of 6 mice developing tumors >300 mm^3^ after 30 days of trametinib treatment. In contrast, at the same time point, none of the tumor-bearing mice treated with trametinib and IgG exhibited tumors larger than 300 mm^3^. Moreover, in mice treated with trametinib +anti-CD8, tumor progression was initiated within 4–5 days, while in the trametinib +IgG group tumor progression started after 20–25 days (figure 2F and online supplemental figure S2I). Depletion efficiency of CD8^+^ T cells was confirmed by flow cytometry for the spleen, blood and tumor of the mice (online supplemental figure S2J) and by IHC staining for CD8^+^ in the tumors on day 30 (online supplemental figure S2K). Co-immunofluorescence (IF) staining of CD8 and PD-1 in vehicle-treated tumors and in tumors that relapsed under trametinib treatment (trametinib +IgG on day 30) showed that CD8^+^ T cells co-expressed PD-1, representing an exhausted phenotype (figure 2G). These findings indicate that CD8^+^ T cells are involved in limiting 4NQO-L progression and promoting the efficacy of trametinib and tumor progression associated with CD8^+^ T cell dysfunction.

### The trametinib/αPD-1 combination results in tumor elimination and immune memory

**Figure 3:**
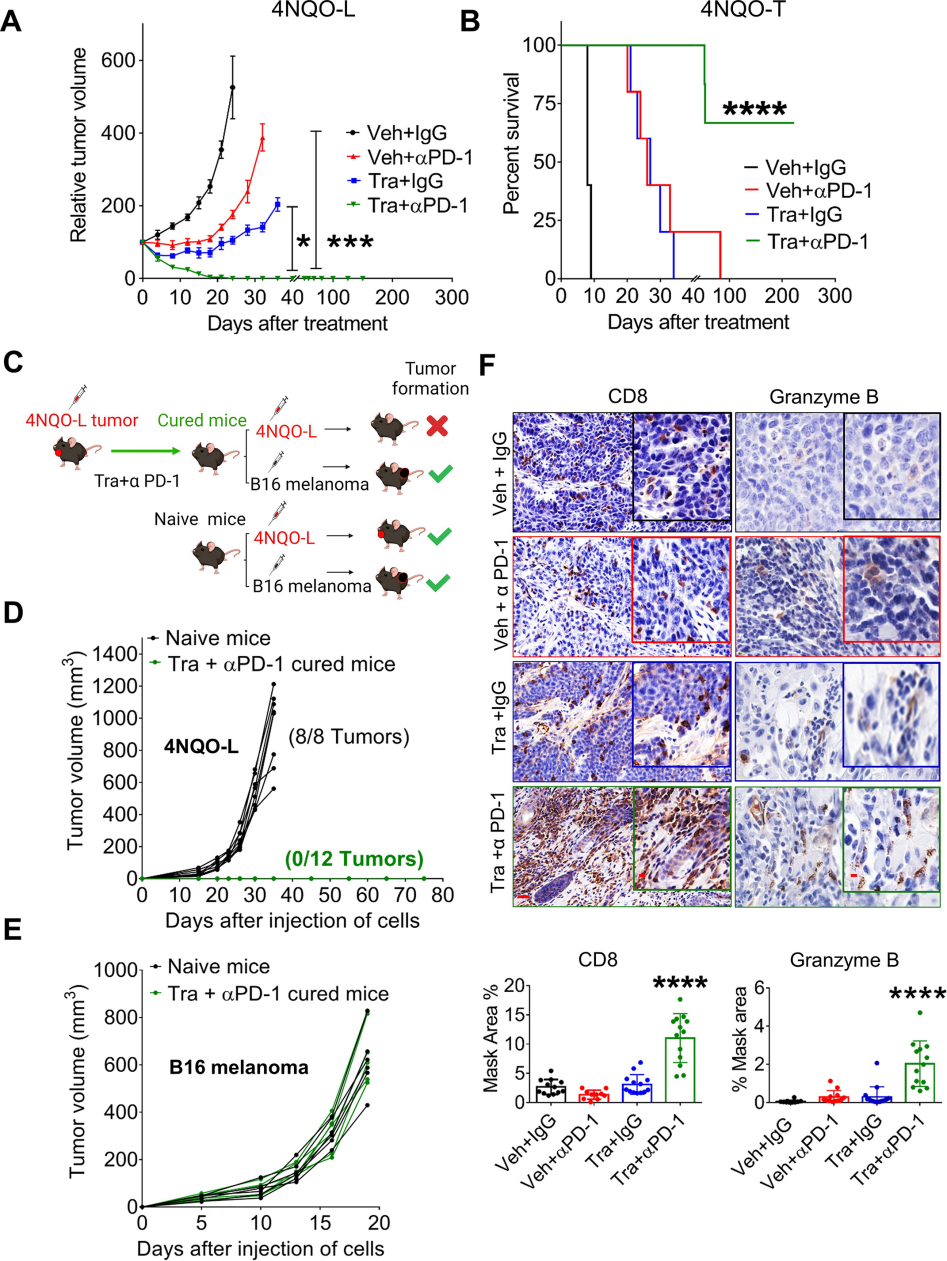
Combination of trametinib and αPD-1 leads to tumor elimination and acquisition of immune memory. (A) Relative tumor volumes of 4NQO-L tumors in WT mice treated with αPD-1, trametinib or the combination of αPD-1 and trametinib. (B) Survival of 4NQO-T-tumor bearing WT mice treated with αPD-1, trametinib, or a combination of αPD-1 and trametinib. (C) Scheme showing the rechallenge experimental setting. (D) Growth curves of 4NQO-L tumors in naïve and cured mice. (E) Growth curves of B16 tumors after injecting naïve and cured mice with B16 melanoma cells. (F) Staining and quantification of CD8^+^ T cells and granzyme B in 4NQO-L tumors treated for 31 days as indicated in online supplemental figure S3E (scale bars: 20 µm; insets 10 µm). One way ANOVA was performed, *p<0.05; ***p<0.001, ****p<0.0001 were considered statistically significant. ANOVA, analysis of variance; Tra, trametinib; Veh, vehicle.

Since CD8^+^ T cells showed an exhausted phenotype in the 4NQO-L tumors, and given that MAPK-pathway mutated HNCs are considered to be ‘hot’ tumors (enriched with CD8^+^ cells) and are thus susceptible to αPD-1 therapy,^4^ we predicted that blocking PD-1 would suppress tumor progression of KRAS-mutated 4NQO-T and 4NQO-L tumors. However, αPD-1 monotherapy had no or minimal antitumor effects in both 4NQO-L and 4NQO-T HNC models, with some mice showing a tumor growth delay and others being completely resistant to the treatment (online supplemental figure S3A). We then posited that further activation of CD8^+^ T cells by supplementing αPD-1 with trametinib would produce superior antitumor activity vs single agents. To explore this premise, we injected 4NQO-L and 4NQO-T cells orthotopically into WT mice. When tumors (3–4 mm diameter) formed, mice were randomized into four groups (vehicle +IgG, vehicle + αPD-1, trametinib +IgG, or trametinib + αPD-1). The 40-day treatment protocol comprised administration of vehicle or trametinib for 5 days, followed by supplementation with IgG or αPD-1 twice a week for an additional 35 days. Analysis of the 4NQO-L tumor volumes and the overall survival of 4NQO-T tumor-bearing mice revealed that treatment with αPD-1 resulted in a transient response that was followed by tumor progression within days. Tumor-bearing mice treated with trametinib exhibited a delay in tumor growth, but within 30 days all mice developed resistance and the tumors progressed (figure 3A and B). However, the trametinib/αPD-1 combination induced a profound and durable antitumor response in both cancer models. In the 4NQO-L tumor-bearing mice, the combination therapy completely eradicated the tumors in all the mice (6/6) (figure 3A). In these animals, tumor relapse was not observed 100 days after completion of the treatment. In the 4NQO-T model, we noted a similar trend with complete elimination of the tumors in 4 out of 6 mice (figure 3B). In the 4NQO-T model, disease relapse occurred in 2/6 mice 40 days after the treatment was stopped, suggesting that prolonged treatment with the combined therapy may have prevented tumor relapse. We further validated the superior activity of therapy combination over single agents in two additional MAPK-driven HNC models, the KRAS^mut^ EpT/C9Ep^31^ and mEERL^37^ (online supplemental figure S3B, C).

To explore whether the mice in which the combined trametinib/αPD-1 treatment had eliminated the tumors retained long-term immune-memory, we rechallenged the animals by injecting 4NQO-L and B16 melanoma cell lines into the right and left flanks, respectively. A control group of naïve mice was similarly injected with 4NQO-L and B16 melanoma cell lines (figure 3C). All naïve mice injected with B16 melanoma and 4NQO-L cells developed measurable tumors. The cured mice rejected the 4NQO-L cells but developed tumors after inoculation with B16 melanoma cells (figure 3D and E, and online supplemental figure S3D). To confirm the involvement of CD8^+^ T cells in tumor elimination, we repeated the same experiment as that described in figure 3A and analyzed some of the tumors before tumor elimination (online supplemental figure S3E, F). Histopathological analysis indicated that the tumor shrinkage induced by the trametinib/αPD-1 combination was associated with a massive infiltration and accumulation of activated CD8^+^ T cells, as determined by granzyme B and CD8 staining (figure 3F). Taken together, these results indicate that activation of CD8^+^ T cells by the trametinib/αPD-1 combination eliminated KRAS-mutated HNC and that treated mice had acquired immune memory.

### Chronic treatment with trametinib prevented sensitization of tumors to αPD-1

**Figure 4:**
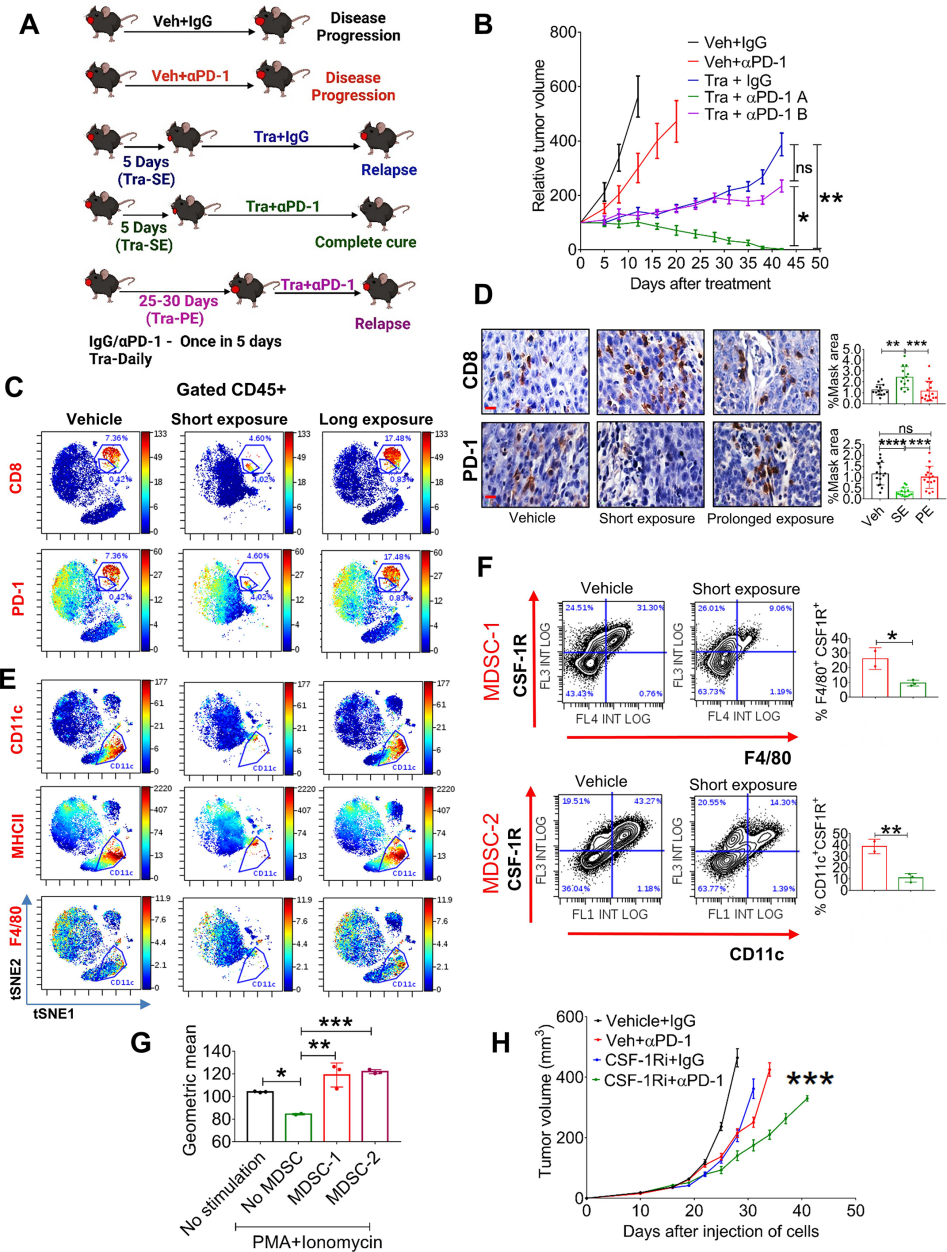
Chronic treatment with trametinib prevents sensitization of tumors to αPD-1. (A) Scheme of the experimental setting. (B) Tumor volumes of 4NQO-L tumors treated as as follows: vehicle +IgG; vehicle + αPD-1; trametinib +IgG; SE of trametinib for 5 days before starting treatment with αPD-1 (Tra + αPD-1 A); and PE of trametinib for 25 days before starting treatment with αPD-1 (Tra + αPD-1 B). (C) viSNE plots of the CyTOF data showing CD8 and PD-1 expression in CD45^+^ cells from 4NQO-T tumors treated with vehicle, (SE; 5 days) trametinib, or a (PE; 33 days) of trametinib. (D) IHC staining (left) of CD8 and PD-1 in 4NQO-L tumors after treatment with vehicle, SE of trametinib, or PE of trametinib (scale bars: 10 µm). Quantification on the right. (E) viSNE plots of the CyTOF data showing CD11c, MHCII and F4/80 expression in CD45^+^ cells from 4NQO-T tumors treated with vehicle, SE of trametinib, or PE of trametinib. (F) Flow cytometric dot plot analysis of a macrophage-like MDSC population (MDSC-1) and a DC-like MDSC population (MDSC-2) in 4NQO-L tumors treated with vehicle or trametinib for 5 days. (G) In vitro proliferation assay of CD8^+^ T cells in co-culture (ratio 1:10) with two cell populations, MDSC-1 and MDSC-2, derived from 4NQO-L tumor-bearing mice. geometric mean fluorescent intensities of CFSC (carboxyfluorescein diacetyl succinimidyl ester) is shown. (H) Tumor growth curves of mice injected with 4NQO-L cells in the lip and treated with veh +IgG, CSF-1R inhibitor (PLX-3397), veh +αPD-1, or combination of CSF-1Ri and αPD-1. one way ANOVA was performed, and *p<0.05, **p<0.01, ***p<0.001, ****p<0.0001 were considered statistically significant. ANOVA, analysis of variance; CSF-1, colony-stimulating factor-1; DC, dendritic cell; IHC, immunohistochemistry; MDSC, myeloid-derived suppressor cells; PE, prolonged exposure; SE, short exposure; Tra, trametinib; Veh, vehicle.

Given that HNC patients can be treated with compounds targeting the MAPK pathway, such as EGFR inhibitors, prior to immunotherapy prompted us to explore how the duration of pretreatment with trametinib influences the sensitivity of tumors to supplementation with αPD-1. Specifically, we tested whether a short exposure (SE) or a prolonged exposure (PE) with trametinib determines the ability of αPD-1 to eliminate HNC tumors. The experimental set-up comprised five groups of 4NQO-L-bearing mice (figure 4A), treated as follows: vehicle +IgG; vehicle + αPD-1; trametinib +IgG; SE of trametinib for 5 days before starting treatment with αPD-1; and PE of trametinib for 25 days before starting treatment with αPD-1. As expected, supplementation with αPD-1 after a SE with trametinib resulted in tumor eradication in all mice. However, supplementation with αPD-1 after a PE with trametinib arrested tumor growth only for a few days, and the tumors started to relapse thereafter (figure 4B). This tumor progression in the trametinib + αPD-1 PE group indicated that trametinib induces a therapeutic window during which αPD-1 efficacy is enhanced, and when mice are exposed to trametinib for an extended period of time, the tumors will regain their αPD-1 resistance phenotype.

To investigate in-depth the effect of trametinib on the heterogeneity of immune cells in the TME of the KRAS-mutated tumors, and to determine whether there is an association between the presence and accumulation of immune cells in the TME and responsiveness to αPD-1, we profiled CD45^+^ cells during trametinib treatment and compared the effect of SE (7 days) vs PE (33 days) of trametinib by using cytometry by time of flight (CyTOF). viSNE analysis of the cytometry data showed that a SE of mice to trametinib resulted in major changes in CD45^+^ cells compared with the vehicle-treated group, while the TME of tumors exposed to trametinib for 33 days had reverted to almost the same landscape as the vehicle-treated group (figure 4C). Analysis of CD8^+^ T cells supported the flow cytometry data presented in figure 2C and online supplemental figure S2F, showing that trametinib treatment induced a reduction of PD-1-positive exhausted CD8^+^ T cells and an increase of activated CD8^+^ T cells (figure 4C). Specifically, the percentage of activated CD8^+^ T cells increased from 0.4% in the vehicle-treated tumors to 4% in the SE trametinib-treated tumors, while the exhausted CD8^+^ T cells were reduced from 7% to 4.6%. In contrast, PE with trametinib induced the accumulation of CD8^+^ T cells expressing PD-1 (figure 4C). We then quantified by IHC analysis the number and activity of CD8^+^ T cells in both cancer models, 4NQO-L and 4NQO-T, after a SE of 5–10 days or a PE of 25–35 days of treatment with trametinib. Tumors treated with trametinib for 5–10 days showed massive infiltration of CD8^+^ T cells into the tumor site compared with the vehicle-treated group (figure 4D and online supplemental figure S4A, B). Notably, in the vehicle-treated tumors we observed a larger amount of CD8^+^ T cells at the tumor edge, while in the trametinib-treated group the infiltrated CD8^+^ T cells also spread inside the tumor bulk (online supplemental figure S4C). Analysis of the PE group (30 days of treatment) showed a reduction in the number of CD8^+^ T cells inside the tumor, and these CD8^+^ T cells regained their exhausted phenotype with upregulation of PD-1 expression (figure 4D and online supplemental figure S4A, B).

Analysis of the myeloid compartment showed that CD11b^+^ cells also underwent major alterations after trametinib administration, with elimination of the CD11b^+^CD11c^+^MHCII^+^PD-L1^+^ subpopulation in the trametinib-treated group (figure 4E and online supplemental figure S4D). The presence of the CD11b^+^CD11c^+^MHCII^+^PD-L1^+^ subset with exhausted CD8^+^ T cells in the TME prompted us to explore whether this CD11b^+^CD11c^+^ population has immunosuppressive capability against CD8^+^ T cells and to determine why a SE with trametinib led to a reduction in the number of these cells. Flow cytometry analysis of the tumors treated with vehicle or trametinib for 5 days showed that the CD11b^+^CD11c^+^MHCII^+^ cells constitute a heterogeneous population expressing CSF-1R^+^ (online supplemental figure S4E). One subpopulation of CSF-1R^+^ cells comprises M2-like macrophages that express high levels of F4/80 and MGL2 (CD103b^+^), and the other population shows a monocytic (dendritic cell) DC-like phenotype with F4/80^negative^ and CD11c^+^^38^ cells (online supplemental figure S4F, G). Quantification of these subsets after 5 days of treatment with trametinib showed a reduction of the M2-like macrophages, namely CSF-1R^+^ CD11b^+^CD11c^+^F4/80^+^ cells (MDSC-1), and of the monocytic DC-like CSF-1R^+^ CD11c^+^MHCII^+^F4/80 cells (MDSC-2) (figure 4F, and online supplemental figure S4F). To test whether these myeloid subpopulations have immunosuppressive effects on CD8^+^ proliferation, we co-cultured sorted MDSC-1 (CD11b^+^CD11c^+^MHCII^+^CSF-1R^+^F4/80^+^) and MDSC-2 (CD11b^+^CD11c^+^MHCII^+^ F4/80^-^CSF^-^1R^+^) cells with naïve CD8^+^ T cells and measured CD8^+^ T cell proliferation in response to stimulation with PMA and ionomycin. The MDSC1 and MDSC2 subsets significantly suppressed the proliferation of PMA/ionomycin-induced naive CD8^+^ T cells (figure 4G). In light of the known role of CSF-1R^+^ cells in αPD-1 efficacy,^39 40^ we tested whether depletion of CSF-1R^+^ cells in the 4NQO-L tumors would be sufficient to enhance αPD-1 efficacy and to eliminate the tumors. To this end, we treated 4NQO-L tumor-bearing mice with the CSF1R inhibitor PLX3397 (pexidartinib) and supplemented the therapy with αPD-1 or IgG (control). The PLX3397 treatment only partially sensitized tumors to αPD-1 as only a tumor growth delay (and not tumor elimination) was detected in these mice (figure 4H). Treatment of mice with PLX3397 was sufficient to reduce the CD11c^+^ population in the tumors, but it did not induce infiltration of CD8^+^ T cells (online supplemental figure S4H). Moreover, treatment with the combination of PLX3397 and αPD-1 resulted in a modest (but significant) infiltration of CD8^+^ T cells compared with vehicle, which induced only a tumor growth delay and not tumor elimination (online supplemental figure S4H), thereby indicating both that elimination of CSF-1R^+^ MDSC is insufficient to potentiate αPD-1 to induce tumor elimination and that trametinib has a broader effect, such as inducing tumor cell growth arrest.

### Tumor-derived CSF-1 determines CD8^+^ T cell activity and therapy efficacy in mice and in patients

**Figure 5:**
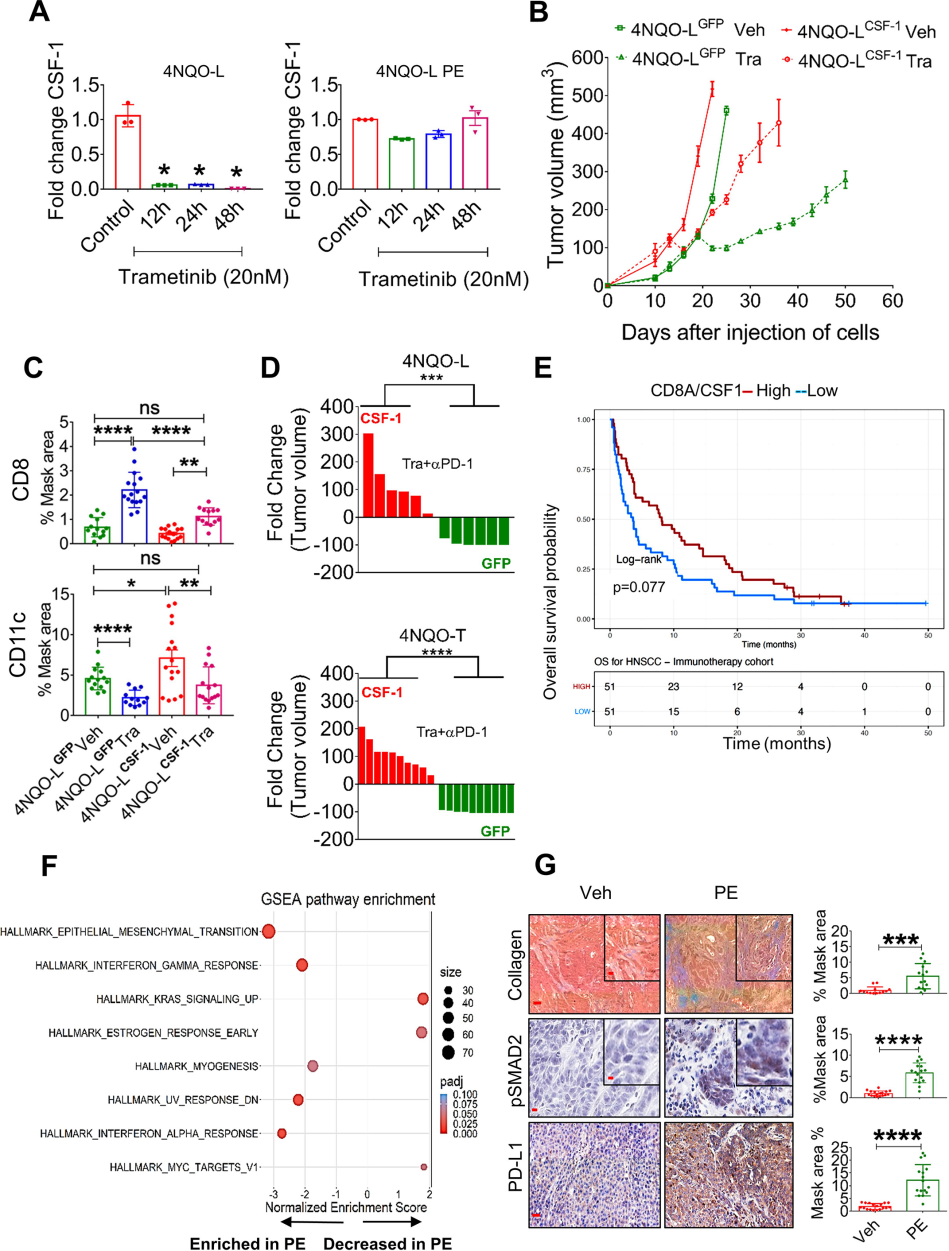
Chronic exposure of trametinib upregulates CSF-1, induces EMT, and prevents trametinib/αPD-1 efficacy. (A) mRNA expression levels of CSF-1 in 4NQO-L (left) and 4NQO-L-PE (right) cells after treatment with trametinib (20 nM) for 0, 12, 24 and 48 hours. (B) Relative volume of 4NQO-L^CSF-1^ and 4NQO-L^GFP^ tumors in WT mice treated with trametinib. (C) Quantification of CD8 and CD11c in 4NQO-L^GFP^ and 4NQO-L^CSF-1^ tumors treated with trametinib for 5 days (SE). (D) Fold change of tumor volumes of 4NQO-L^GFP^ and 4NQO-L^CSF-1^ and 4NQO-T^GFP^ and 4NQO-T^CSF-1^ tumors treated with the combination of trametinib and αPD-1. (E) Overall survival (OS) curves for 102 patients with head and neck squamous cell carcinoma (HNSCC) treated with αPD-1/PD-L1 (CLB-IHN cohort) and according to high vs low values of the CD8A/CSF-1 ratio. Survival distributions were estimated using the Kaplan-Meier method and compared by the log-rank test between two groups. Patients were binarized at the median. (F) GESA analysis of RNAseq of 4NQO-L, 4NQO-L-PE, 4NQO-T, and 4NQO-T-PE cells. (G) Collagen (trichrome), pSMAD2 (IHC), and PD-L1 (IHC) in 4NQO-L tumors treated with vehicle or PE of trametinib (scale bars: 100 µm (inset 20 µm), 50 µm (inset 10 µm), and 100 µm respectively). For statistics, an unpaired two-sided t-test or one-way ANOVA was performed. *P<0.05; **p<0.01; ***p<0.001, ****p<0.0001 were considered statistically significant. ANOVA, analysis of variance; CSF-1, colony-stimulating factor-1; EMT, epithelial to mesenchymal transition; IHC, immunohistochemistry; PE, prolonged exposure; SE, short exposure; Tra, trametinib; Veh, vehicle.

To explore how trametinib treatment reduces the number of CSF-1R^+^ cells and increases the infiltration ability and activation of CD8^+^ T cells, we initially used a publicly available database of single-cell RNA sequencing of HNC patients to examine whether CSF-1, the ligand of CSF-1R, is expressed by tumor cells. Analysis of a cohort of 9 HNC patients showed that some tumor cells, primarily those belonging to the basal and the atypical subsets, express CSF-1 (online supplemental figure S5A).^41^ We then explored whether trametinib treatment affects the transcriptional levels of CSF-1 in tumor cells. qPCR analysis of 4NQO-L cells treated with trametinib for 12, 24 and 48 hours showed decreased CSF-1 levels in vitro (figure 5A). However, when 4NQO-L cells were exposed to trametinib for several weeks, no reduction of CSF-1 was detected. We then overexpressed CSF-1 in epithelial 4NQO-L cells and evaluated the tumor response to trametinib in vivo, including profiling the number of MDSCs expressing CD11c^+^ and CD8^+^ T cells in the tumor. The expression level of CSF-1 by the overexpressing cells, 4NQO-L^CSF-1^, was three times higher than that of the control cells expressing GFP (4NQO-L^GFP^) (online supplemental figure S5B). Analysis of the tumor growth in the control WT mice showed that 4NQO-L^CSF-1^ cells grew slightly faster than 4NQO-L^GFP^ cells, but more significant differences in tumor volume were observed after trametinib treatment. While trametinib treatment induced tumor growth arrest for 20 days in 4NQO-L^GFP^ tumor-bearing mice, rapid tumor progression was observed in 4NQO-L^CSF-1^ tumor-bearing mice (figure 5B). Notably, no differences in the sensitivity of the two cell lines to trametinib treatment were observed in vitro (online supplemental figure S5C). IHC analysis of tumors after 5 days of treatment with trametinib showed massive infiltration of CD8^+^ T cells into 4NQO-L^GFP^ tumors, but this infiltration was attenuated in 4NQO-L^CSF-1^ tumors, and significantly fewer CD8^+^ T cells were present in trametinib-treated 4NQO-L^CSF-1^ tumors compared with 4NQO-L^GFP^ tumors (figure 5C, online supplemental figure S5D). Moreover, the number of CD11c^+^ cells was higher in 4NQO-L^CSF-1^ tumors than in 4NQO-L^GFP^ tumors, while in the 4NQO-L^CSF-1^ tumors treated with trametinib the number of CD11c^+^ cells remained as high as the number in 4NQO-L^GFP^ tumors (figure 5C, online supplemental figure S5D). Based on the negative association between CSF-1 and infiltration of CD8^+^ T cells, we assumed that CSF-1 is a critical factor in regulating the CSF-1R^+^CD11c^+^ MDSC cells in the TME and can thus prevent tumor elimination mediated by the trametinib/αPD-1 combination. To test this hypothesis, we injected 4NQO-L^GFP^ and 4NQO-L^CSF-1^ cells into WT mice and compared the efficacy of the trametinib/αPD-1 combination in eliminating tumors in the two groups of mice. Indeed, we observed that combined trametinib/αPD-1 therapy led to the shrinking of the 4NQO-L^GFP^ tumors, and in 5 out of the 6 mice tumors were eliminated within 35–41 days (figure 5D and online supplemental figure S5E). In contrast, following administration of the combined trametinib/αPD-1 therapy to mice bearing the 4NQO-L^CSF-1^ tumors, 3 out of the six tumors progressed, and three showed stable disease (figure 5D and online supplemental figure S5E). Similar efficacy studies were observed when we tested the response of therapy combination of trametinib/αPD-1 in 4NQO-T^GFP^ and 4NQO-T^CSF-1^ tumors (figure 5D and online supplemental figure S5B, E). These results indicate that CSF-1 served as a key player in modulating CSF-1R^+^CD11c^+^ MDSCs in the TME, thereby preventing the tumor elimination induced by αPD-1 supplementation.

To explore whether these findings can serve as indicators of response to immunotherapy, we undertook gene expression profiling of 102 confirmed recurrent HNC patients pretreated with αPD-1/αPD-L1 before the initiation of immunotherapy at Center Leon Berard (Lyon France) (online supplemental table S5). By calculating the ratio of the expression of CD8A to the expression of CSF-1, we found that a high CD8A/CSF-1 ratio in pretreated patients led to a higher overall survival (binarization at median; p=0.077) with HR of 0.69; 95% CI 0.46 to 1.04 (figure 5E). Consistently, a high CD8A/CSF-1 ratio also tended to be associated with a clinical benefit in response to immunotherapy (defined as complete response, partial response, and stable disease for at least 6 months; p=0.077) (online supplemental figure S5F)). A similar trend was observed in patients with lung cancer, as responders to immunotherapy expressed higher ratio levels of CD8A/CSF-1 than non-responders (p=0.052) (online supplemental figure S5G).^42^

To seek an explanation for the constancy of CSF-1 levels in cells that were chronically exposed to trametinib (figure 5A), we compared the whole transcriptome of 4NQO-L and 4NQO-T cells before and after a PE to trametinib (online supplemental table S6). While we did not observe differences in baseline levels of CSF-1, upregulation of genes of the epithelial to mesenchymal transition (EMT) signature were significantly enriched after PE to trametinib (figure 5F). Specifically, EMT-associated protein expression, such as VIM, TGFβ2, COL1A1, and PD-L1, was upregulated (online supplemental table S7). IF and western blot analysis confirmed an EMT shift in cellular plasticity and upregulation of PD-L1 in vitro (online supplemental figure S5H, I). An in vivo comparison of 4NQO-T and 4NQO-L tumors treated with vehicle or trametinib for 25–30 days (PE) showed an increase in PD-L1, pSMAD2, and collagen expression within the TME in the trametinib-treated tumors compared with the vehicle-treated group (figure 5G and online supplemental figure S5J). We confirmed the association of expression of the EMT genes, *VIM* and *ZEB1*, with CSF-1 in the TCGA dataset of HNC cancer patients (online supplemental figure S5K). We then explored the transcription factors (TFs) that may maintain CSF-1 expression in 4NQO-PE cells. To this end, we used the oPOSSUM.3 system^43^ to perform TF analysis for the genes upregulated in PE cells (FC<-1, Padj <0.001) and found that 65 TFs were activated (Z-score >2) (online supplemental table S8). Cross-section analysis of these TFs with a list of TFs known to bind the CSF-1 promoter extracted from the ENCODE Chip-seq (online supplemental figure S5L) showed that AP1, STAT1/3, CTCF, and TBP might be implicated in CSF-1 expression (online supplemental figure S5L). Among these five TFs, STAT1 and STAT3 expression levels showed the highest correlation with CSF-1 levels, and knock down of STAT3 reduced CSF-1 expression in 4NQO-L-PE cells (online supplemental figure S5M, N). Taken together, our findings show that prolonged treatment of KRAS-mutated HNC with trametinib resulted in, signaling and transcriptional adaptation, leading to EMT and STAT pathway activation and maintainance of CSF-1 levels in the presence of trametinib, thereby prevents the efficacy of supplementation with αPD-1.

## Discussion

**Figure 6:**
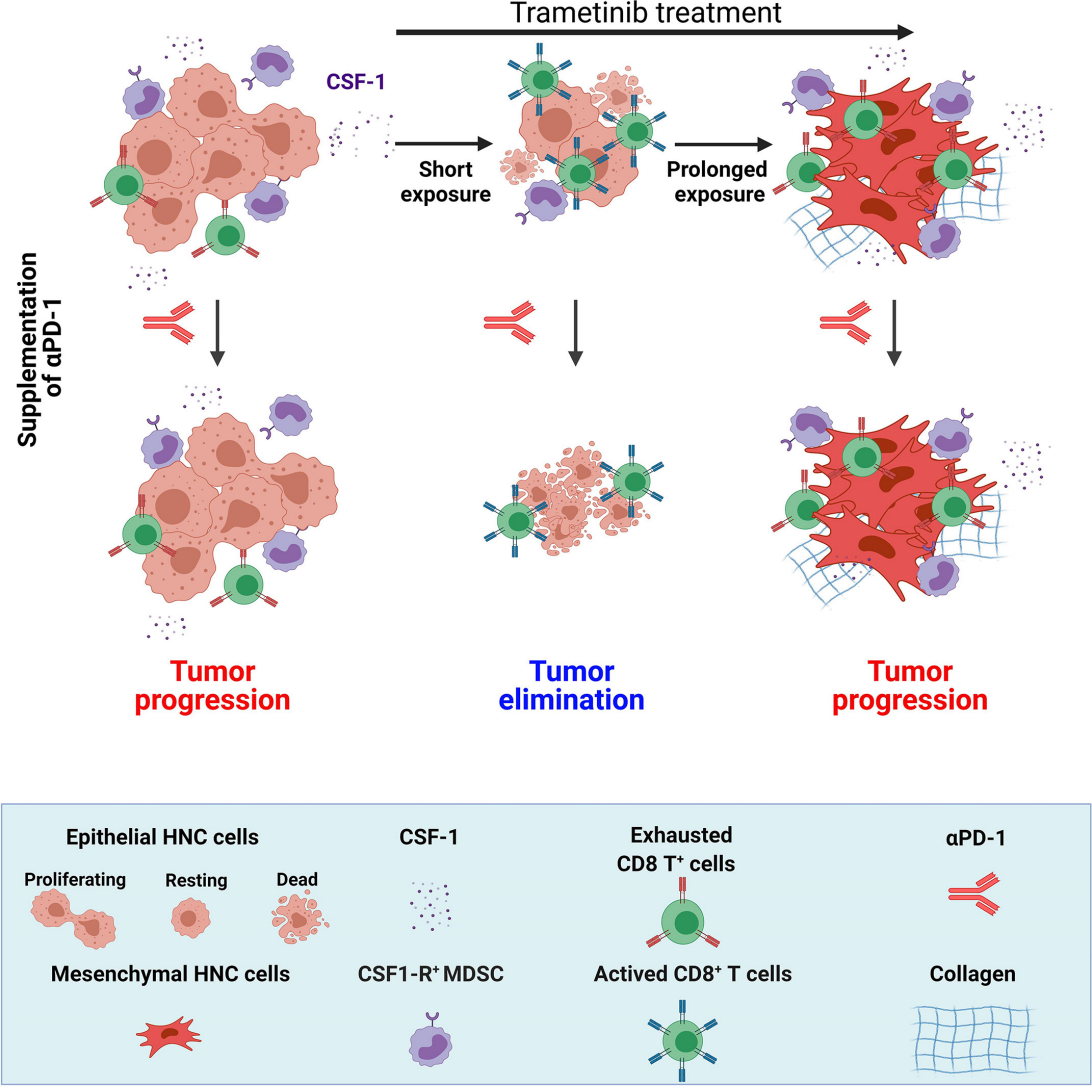
The effect of the duration of trametinib treatment on sensitization of MAPK-pathway mutated HNC to supplementation of αPD-1. MAPK-pathway mutated HNC are resistant to αPD-1 and sensitive to trametinib. Short exposure (SE) of trametinib leads to the reduction of CSF-1 secretion, attenuation of CSF-1R^+^ MDSCs and infiltration of active CD8^+^ T cells; while prolonged exposure (PE) of trametinib induced EMT phenotype and restored the CSF-1 secretion, CSF-1R^+^ MDSCs and exhaustion of CD8^+^ T cells. Supplementation of αPD-1 after SE of trametinib leads to complete elimination of tumor but supplementation of αPD-1 after PE of trametinib resulted in tumor progression. (Scheme was created with BioRender.com). CSF-1, colony-stimulating factor-1; EMT, epithelial to mesenchymal transition; HNC, head and neck cancer; MAPK, mitogen-activated protein kinases; MDSC, myeloid-derived suppressor cells.

In this study, we demonstrated that trametinib treatment delayed the initiation and progression of MAPK-pathway-mutated HNC and changed the heterogeneity of the immune cells in the TME, which subsequently determined the susceptibility to trametinib/αPD-1 combination therapy. In the immediate response to a short treatment with trametinib, defined as a SE of ~5–10 days, tumors were enriched with activated CD8^+^ T cells. However, after prolonged treatment (PE of ~>25 days), tumors adapted to the therapeutic stress and re-established an immunosuppressive environment enriched with exhausted CD8^+^ T cells. Importantly, activation of CD8^+^ T cells with αPD-1 after a SE of trametinib resulted in tumor elimination and the establishment of immune memory, while supplementation with αPD-1 after a PE of trametinib resulted in tumor progression (figure 6).

Several studies in preclinical models and in patients with cancer have demonstrated that targeting the MAPK pathway (ie, with RAF and MEK inhibitors) induces CD8^+^ T cell infiltration into tumors and improved immunotherapy efficacy.^26 44–47^ Mechanistically, MAPK pathway inhibition has a dual effect: on the one hand, it downregulates the expression of immunosuppressive factors such as IL-8 and IL-1 and increases the infiltration of activated CD8^+^ T cells, but on the other hand, it counteracts immune activation through upregulation of PD-L1 by tumor cells. Similarly, in our KRAS-mutated HNC tumors, trametinib treatment initially induced temporary immune activation with massive CD8^+^ T cell infiltration and reduced the numbers of CSF-1R^+^CD11c^+^ MDSCs, but chronic treatment resulted in immune suppression. The reduction in the accumulation of the MDSCs expressing CSF-1R^+^CD11c^+^ seems to be mediated, at least in part, by a reduction in tumor-derived CSF-1. Indeed, ectopic CSF-1 overexpression by tumor cells attenuated the trametinib-induced reduction of CSF-1R^+^CD11c^+^ MDSCs and the infiltration of activated CD8^+^ T cells into the TME. CSF-1 is a key protumorigenic chemokine that promotes the accumulation, differentiation, and proliferation of subsets of CSF-1R^+^ MDSCs that suppress the activation and proliferation of T cells.^48–50^ Several reports have shown that high expression of CSF-1 is associated with the suppression of T cells and with resistance to immunotherapies, such as αPD-1.^39 40 51^ In line with these observations, depletion of CSF-1R^+^ cells with small molecule inhibitors attenuates tumor progression^52^ and enhances therapy efficacy.^39 53^ However, while the suppressive activity of CSF-1R^+^ cells toward CD8^+^ T cells and natural killer cells has been reported in multiple cancers,^54–57^ targeting CSF-1R^+^ cells in mice bearing 4NQO-L tumors had only a minor effect on tumor growth.

Allegrezza *et al*^28^ have shown that trametinib reduces MDSC myelopoiesis and exhibits enhanced efficacy against KRAS-driven breast cancer due to the activation of CD8^+^ T cells. Our results are in line with these findings, as trametinib was more potent in immunocompetent (WT) than in immunocompromised (NSG) mice, and depletion of CD8^+^ T cells attenuated the efficacy of trametinib in WT mice. Using 4NQO-L^CSF-1^ and 4NQO-L^GFP^ tumors we provided further evidence to support the influence of tumor-derived CSF-1 on CSF-1R^+^CD11c^+^ MDSCs and CD8^+^ T cells in response to therapy in vivo. We showed an association between trametinib-induced down-regulation of CSF-1 expression and a reduction in CD11c^+^CSF-1R^+^ MDSCs with the sensitivity of the tumors to αPD-1. However, depletion of CSF-1R^+^CD11c^+^ cells with the CSF-1R inhibitor PLX3397 in combination with αPD1 only induced a tumor growth delay and was not able to eliminate the tumors. These results differ from reports showing that CSF-1R^+^ elimination can enhance the efficacy of immunotherapy and cause tumor elimination in other cancer types.^51 57^ The minor effect of PLX3397 compared with trametinib in sensitizing tumors to αPD-1 highlights the fact that downregulating CSF-1 is only a part of the pleiotropic role played by trametinib in anti-cancer immunity. This notion is reinforced by the evidence of trametinib-induced growth arrest of KRAS-mutated tumors, accompanied by CD8^+^ T cell infiltration into the tumors and by reprogramming CD8^+^ T cells into memory stem cells with potent antitumor effects.^22 27 28^

One of the key findings of our study is the demonstration of a therapeutic vulnerability induced by trametinib treatment that enables αPD-1 to facilitate HNC eradication in mice. The induction of immune activation following treatments with targeted therapy, chemotherapy, or irradiation to enhance immunotherapy efficacy has been described in HNC patients.^58^ Recently, Choi *et al* showed that pulsatile treatment of KRAS-mutated tumor-bearing mice with selumetinib or trametinib had a greater effect on immune activation and susceptibility to combination therapy with CTLA4 as compared with continuous treatment.^27^ These results further support the importance of induction by anti-MEK1/2 therapies of a potent and prolonged immune activation.

In our study, as well as in others, the immune activation mediated by trametinib is associated with a delay of tumor growth and, when therapy is subsequently initiated, it is also associated with immune suppression.^19 59^ Explanations for our findings may be drawn from previous reports showing that prolonged treatment with trametinib induces a reduction in CD8^+^ T cells in the tumors^17^ and a signaling adaptation in HNC cells related to the mesenchymal phenotype, which may be promoted by the TF, YAP.^60 61^ It is also known that tumors showing an EMT phenotype are enriched with immunosuppression characteristics.^62 63^ For example, tumor cells undergoing EMT upregulate immunomodulatory surface proteins, such as PD-L1, and express high levels of chemokines that regulate immune suppressive factors, such as IL-10, TGF-β and CSF-1.^64 65^ Thus, a potential explanation for the constancy of CSF-1 expression in PE tumor cells is the hyperactivation of TFs in EMT-like cells, such as TWIST-1, which has been shown to maintain CSF-1 expression,^66^ or via activation of STAT3 as we showed in figure 5 and online supplemental figure S5. Another mechanism that further impairs αPD-1 efficacy in tumors undergoing EMT is the upregulation of collagen, which has been shown to induce CD8^+^ T cell exhaustion and to promote resistance to αPD-1/PD-L1.^67 68^

Recently Hass *et al*^69^ demonstrated that the acquisition of resistance to targeted anti-MAPK therapy (with MEK or RAF inhibitors) conferred cross-resistance to immunotherapy in melanoma and they proposed that immunotherapy should be administrated before patients develop resistance to anti-MEK1/2. Their basic observation that prolonged treatment with anti-MEK1/2 resulted in resistance to αPD-1 (also shown in our HNC models) is suggestive of a general phenotype of MEK inhibitors in MAPK-pathway mutated tumors. Nevertheless, the mechanisms may be different in the two sets of experiments: while Hass *et al* showed that treatment of tumor-bearing mice with anti-MAPK increased the number of CD103^+^ DC cells in the TME, thereby activating CD8^+^ T cells, we proposed an alternative mechanism based on the elimination of the immune-suppressive CD11c^+^/CSF-1R^+^ cells.

Sequential treatment with immunotherapy and targeted therapies appeared to be important for optimal antitumor efficacy. Recently, Wang *et al*^70^ showed that pretreatment with immunotherapy before supplementation with anti-MEK maximized therapy effectiveness and even reduced brain metastasis. Mechanistically, such a treatment sequence affected the TME by reducing M2-like tumor-associated macrophages, which facilitate better clonal expansion and persistence of tumor-specific CD8^+^ T cells.

HNCs are classified into four major subtypes based on RNA sequencing.^41 71^ MAPK-pathway mutated tumors are associated with the immune subtype that is enriched with CD8^+^ T and CD11c cells,^4^ while tumors that have hyperactivated MAPK pathway via RTK stimulation are associated with the basal subtype.^3^ Our observation that tumors became sensitive to αPD-1 only when trametinib reduced CSF-1 expression and induced CD8^+^ infiltration encouraged us to explore whether the ratio between CSF-1 and CD8A is associated with a clinical benefit derived from administering αPD-1 to HNC patients. An analysis of gene expression profiles from preimmunotherapy biopsies of HNC patients showed that patients expressing low levels of CSF-1 and high levels of CD8A displayed a better overall survival and an association with a clinical benefit of αPD-1 and αPD-L1 therapies. This analysis does not distinguish between tumor-derived and stromal-derived CSF-1, and the role of the endogenous tumor-derived CSF-1 requires further investigation.

In conclusion, our findings show the potential of sensitizing MAPK-pathway mutated HNC to αPD-1 by pretreating patients with MAPK inhibitors, and then continuing with the therapy combination. Pretreatment with trametinib interfered with the interaction between the malignant cells and the microenvironment via reducing tumor-derived CSF-1. Downregulation of CSF-1 expression and arrest of tumor cell proliferation resulted in an immune-active TME, which is required for immunotherapy efficacy. Such an active immune state of the TME defined by the CD8A to CSF-1 expression ratio is associated with a positive response to αPD-1/αPD-L1 therapies in patients with cancer.

## Data Availability

Data are available in a public, open access repository. Data are available on reasonable request. Raw RNA-seq data is deposited at the BioProject ID PRJNA795864.

## Data availability statement

Raw RNA-seq data is deposited the BioProject ID PRJNA795864. All data are available in the main text or supplementary materials. Any data and materials that can be shared will be released via a material transfer agreement. Raw RNA-seq data is deposited at the BioProject ID PRJNA795864.
