# Supplementary material for: MEK1/2 inhibition transiently alters the tumor immune microenvironment to enhance immunotherapy efficacy against head and neck cancer
Source: J Immunother Cancer. 2022 Mar 15;10(3):e003917. doi: 10.1136/jitc-2021-003917 (PMC8928405; doi:10.1136/jitc-2021-003917)
Supplement: Supplementary data [file jitc-2021-003917supp001.pdf]

MATERIALS AND METHODS

Genomic analysis of MAPK pathway mutated genes. The MAPK pathway genes were defined in the CBbioPortal [1–4]. Whole-exome sequencing data for MAPK pathway-specific mutation was retrieved from TCGA, GENIE, and ICGC for mutational analysis on 9 February 2021 (www.cbioportal.org, www.genie.cbioportal.org, www.dcc.icgc.org).

MAPK pathway activation - TCGA analysis. A heatmap was generated with the web tool ClustVis [5]. MAPK pathway activity was inferred using the progeny R package [6], as described previously [7]. GSVA scores were computed with the GSVA package [7] in RStudio (4.0.2) based on selected gene sets from the Molecular Signatures Database (MSigDB [8,9]).

Oral carcinogenesis with 4-nitroquinoline N-oxide (4NQO). C57BL/6J mice, 4–5 weeks old, purchased from the Envigo laboratory, were used for the 4NQO carcinogenesis studies. A stock solution of 4NQO (N8141 Sigma, St. Louis, MO) in propylene glycol was prepared weekly at a concentration of 5 mg/mL and administered at a concentration of 50 µg/mL in the drinking water for 12 weeks. The development of tumors in the oral cavity of the mice was examined weekly, and at end of the experiment, tumors were collected for analysis. For the survival experiments involving trametinib (GSK1120212, JTP-74057), the drug was given after 12 weeks of 4NQO treatment (trametinib was dissolved in 4% DMSO and then diluted in corn oil and administered at a dose of 1 mg/kg/day).

In vivo experiments. Mice were housed in air-filtered laminar flow cabinets with a 12-h light/dark cycle and supplied with food and water ad libitum. All animal experiments were carried out under the Institutional Animal Care and Use Committee (IACUC) of Ben-Gurion University of the Negev (BGU’s IACUC) according to specified protocols to ensure animal welfare and to reduce suffering. The animal ethical clearance protocol numbers used for the study are IL-80-12-2015 (E), and IL-29-05-2018 (E). In vivo experiments were conducted using

6- to 8-week-old NSG mice (NOD.Cg-Prkdcscid Il2rgtm1Wjl/SzJ, Jackson Labs) and C57BL/6J (WT) mice (Envigo, Huntingdon, UK, C57/BL/6).

Patient-derived xenografts (PDXs). For this study, we used two PDXs, designated SE19 (PDX-1) and SE103 (PDX-2), previously established in our lab [10,11]. The tumors were transplanted into the right and left flanks of NSG mice for the trametinib (1 mg/kg/day) efficacy experiments. In all experiments, tumor measurement was performed with a digital caliper, and tumor volumes were determined according to the formula: length × width^2 × π/6.

Immunohistochemistry. Tissues were fixed in a 4% paraformaldehyde (PFA) solution for a maximum of 24 h at room temperature, dehydrated, and embedded in paraffin. The tissue sections were de-paraffinized with xylene. H_2O_2, 3%, was used to block the endogenous peroxidase activity for 20 min, and thereafter the sections were rinsed in water for 5 min. Antigen retrieval was performed in citrate buffer (pH 6) at 99.99 °C for 15 min. Sections were then blocked for 1 h at room temperature with blocking solution [phosphate buffered saline (PBS), 0.1% Tween, 5% bovine serum albumin (BSA)], followed by incubation with primary antibody (diluted in blocking solution) overnight at 4°C. A list of antibodies used is given in Supplementary Table S9. The ABC kit (VECTASTAIN Cat. VE-PK-6200) was used for color detection according to the manufacturer's protocol. Sections were counter-stained with hematoxylin and mounted in mounting medium (Micromount, Leica Cat. 380-1730). Slides were scanned using the Pannoramic Scanner (3DHISTECH, Budapest, Hungary) and analyzed with a QuantCenter (3DHISTECH, Budapest, Hungary).

Cell lines. 4NQO-L and 4NQO-T, KRAS^{mut} EpT/C9Ep murine cell lines developed as described previously [12][13].B16 melanoma and And all other murine cell lines were maintained at 37 °C in a humidified atmosphere at 5% CO_2 in DMEM supplemented with 1% L-glutamine 200 mM, 100 units each of penicillin and streptomycin, and 10% fetal bovine serum (FBS). mEERL cells were kindly provided by Dr. Paola D. Vermeer (Sanford Research, South Dakota,USA).

Prasad M, *et al. J Immunother Cancer* 2022; 10:e003917. doi: 10.1136/jitc-2021-003917

The cells were grown in Dulbeco's modified Eagle's medium containing 10% fetal bovine serum, 22% Ham's F12, 1% pen/strep, 25 μg/mL hydrocortisone, 5 μg/mL transferrin, 5 μg/mL insulin, 1.36 ng/mL tri-iodo-thyronine, and 5 ng/mL epithelial growth factor [14][15].

Cells were routinely tested for mycoplasma infection and treated with appropriate antibiotics if needed (De-Plasma, TOKU-E, D022). For generating 4NQO-L-PE and 4NQO-T-PE cells, 4NQO-L and 4NQO-T were exposed to 50–100 nM of trametinib for several weeks.

IC_{50} assay. Cells were seeded in 96-well plates (5000 cells/well), treated with increasing concentrations of trametinib, and allowed to proliferate for 72 h. Cells were then stained with crystal violet (1 g/L) for 10 min, rinsed, and dried, and bound crystal violet was dissolved out with 10% acetic acid. Absorbance was measured at 570 nm (BioTek™ Epoch™ spectrophotometer). The dose-response curve was plotted, and IC_{50} values were calculated using GraphPadPrism7 software.

Western blotting. Cells were harvested and lysed using lysis buffer (50 mM HEPES, pH 7.5, 150 mM NaCl, 1 mM EDTA, 1 mM EGTA, 10% glycerol, 1% Triton X-100, 10 μM $MgCl_2$), supplemented with phosphatase inhibitor (Bio tool, B15001A/B) and protease inhibitor (Millipore Sigma, P2714-1BTL) cocktails, and placed on ice for 30 min, followed by 3 min of sonication. Lysates were cleared by centrifugation (15 min, 14,000 rpm, 4 °C). Supernatants were collected, and whole-cell lysates (25 μg) were separated on 10% SDS–PAGE and blotted onto PVDF membranes (BioRad Trans-Blot® Turbo™ transfer pack #1704157). Membranes were blocked for 1 h in blocking solution [5% BSA (Amresco 0332-TAM) in Tris-buffered saline (TBS) with 0.1% Tween] and then incubated with primary antibodies diluted in blocking solution. Mouse and rabbit horseradish peroxidase (HRP)-conjugated secondary antibodies were diluted in blocking solution. Protein-antibody complexes were detected by chemiluminescence [Westar Supernova (Cyanagen Cat. XLS3.0100) and Westar Nova 2.0 (Cyanagen Cat. XLS071.0250)], and images were captured using the Azure C300 Chemiluminescent Western

Blot Imaging System (Azure Biosystems). Details of antibodies used are presented in Supplementary Table S9.

H&E staining. Rehydrated tissues were stained with hematoxylin for 3 min, washed with tap water for removing excess stain, and stained with eosin for 30 s. Excess stain was removed by dipping in 70% ethanol. Tissues were then dehydrated and mounted.

Immunofluorescence. For IF monitoring, cells were seeded on 24-mm round coverslips. Cells were washed with PBS and fixed in 4% PFA for 30 min at room temperature. Cells were rinsed with PBS, followed by permeabilization using 0.05% Triton X-100 (Millipore Sigma) for 10 min in PBS. Antigen retrieval for tissue section staining was performed in citric acid. Then, cells/tissues were blocked using a blocking solution (5% BSA in TBS-T) for 1 h at room temperature. Cells/tissues were incubated with primary antibodies overnight at 4 °C, washed in PBS-T and stained with secondary antibodies. Slides were mounted with DAPI Fluoromount-G® (Southern Biotech, Birmingham, MA, USA, 0100-20).

Masson's Trichrome Staining. Tissues were rehydrated and stained using Masson's Trichome Staining Kit as per the manufacturer's instructions (Bio-optica- 04-01802). Slides were mounted and scanned using the Pannoramic Scanner (3DHISTECH, Budapest, Hungary) and analyzed with a QuantCenter (3DHISTECH, Budapest, Hungary).

OPAL multiplexed staining of tissues. Tumor tissues were prepared for staining as described above, and then IF was performed as described in the manufacturer's protocol (Opal™ 4-Color Manual IHC Kit, cat no. NEL810001KT). Briefly, tissues were incubated with an anti-CD8 antibody (Cell Signaling Technology, 1:500) for 1 h at room temperature. Tissues were then washed with Tween-PBS and incubated with rabbit horseradish peroxidase (HRP)-conjugated secondary antibody (Jackson ImmunoResearch, PA, USA, 1:500) for 1 h at room temperature. Thereafter, tissues were again washed and stained with FP1487001KT [Opal™ 570 FP1488001KT (red)], according to the manufacturer’s protocol. The method is based on

stripping the primary and secondary antibodies (not reducing fluorescent signals) and then restaining with a second primary antibody, using the same procedure. After stripping, the tissues were incubated with α-PD-1 antibody (Cell Signaling Technology, 1:250) for 1 h at room temperature, washed, and stained again with rabbit HRP-conjugated secondary antibody, followed by staining with FP1487001KT. Slides were mounted with DAPI Fluoromount-G® (SouthernBiotech, Birmingham, MA, USA, 0100-20). IHC and IF slides were scanned using the Pannoramic Scanner (3DHISTECH, Budapest, Hungary) and analyzed with a QuantCenter (3DHISTECH, Budapest, Hungary) using a single threshold parameter for all images with a specific staining in each experiment.

Orthotopic models for drug efficacy/survival studies. Orthotopic models were established by injecting tumor cell lines into the lips or tongues of the mice. For the efficacy studies, treatments were started when the tumors in the lip had reached 4–5 mm in diameter. Tumors were measured with a digital caliper twice a week. At the end of the experiment, animals were sacrificed, and the tumors were collected. For survival experiments, cells were injected orthotopically into the tongues of the mice. For efficacy experiments with trametinib and αPD-1 antibody, trametinib was used at 1 mg/kg/day, and αPD-1 (rat anti-mouse PD-1, BE0146-25 -clone RMP1-14) and IgG (rat anti-mouse IgG2a, BE0089-25) from Bio X Cell were used at a concentration of 100 µg/mouse. For the efficacy experiments with the CSF-1R inhibitor, pexidartinib (Chemscene #4256), the compound was used at a concentration of 75 mg/kg/d dissolved in DMSO (4%), and corn oil was used as the vehicle.

CD8 depletion experiment. In vivo Plus™ anti-mouse CD8α (rat anti-mouse CD8α, BP0061-25 Clone 2.43) or IgG In vivo Plus ™ rat IgG2b isotype control, both from Bio X Cell, were used for the CD8 depletion experiments. The animals were given IP, 1 mg/mouse of αCD8 antibody or IgG before 2 days of trametinib treatment, and treatment was continued with 500 µg/mouse of αCD8 antibody or IgG every 5 days.

Prasad M, *et al. J Immunother Cancer* 2022; 10:e003917. doi: 10.1136/jitc-2021-003917

CyTOF. *Sample preparation.* For CyTOF analysis, 4NQO-T tumors were isolated from mice treated as indicated (Vehicle, Short exposure and Prolong exposure), and pools of 6-8 tumors were analysed for each condition. The tumor tissue was washed with PBS, cut into small pieces, and suspended in 1 mg/mL type II collagenase diluted in RPMI 1640 medium. The gentleMACS™ Octo Dissociator (Miltenyi Biotec, Bergisch Gladbach, Germany) was used to dissociate the tumor tissue at 37 °C for 35 min. Then, the samples were filtered through a 40-μm strainer to obtain a single-cell suspension. Erythrocytes were removed with ACK lysing buffer [0.15 M NH_4Cl, 10 mM $KHCO_3$, and 0.5 M EDTA (pH 7.2–7.4)]. Cells were then washed once with cell staining medium (CSM; PBS, 2% BSA and 0.07% azide), and samples were resuspended in 500 μL of Cell-ID Intercalator-103Rh (1:2000) (Fluidigm) for 15 min at room temperature or overnight at 4 °C. The cells were washed in CSM and resuspended in 50 μL of CSM. Most antibodies were obtained pre-conjugated to heavy-metal isotopes from Fluidigm. Cell-surface antibody master mix was prepared by adding appropriate dilutions of all cell-surface antibodies into 50 μL of CSM per sample. The antibody master-mix was then filtered through a pre-wetted 0.1-μm spin-column (Millipore) to remove antibody aggregates, and 50 μL of this filtrate were added to the sample resuspended in 50 μl of CSM. After incubation for 60 min at room temperature, cells were washed once with CSM and fixed in 500 μL of 1.6% PFA in PBS and stored at 4 °C. Cells were washed once with CSM and resuspended in intercalation solution [1.6% 0.5 μM iridium-intercalator (Fluidigm)] for 20 min at room temperature or overnight at 4 °C.

Data acquisition. Before data acquisition, samples were washed once in CSM and twice in doubly distilled water and filtered through a cell strainer (Falcon). Cells were then resuspended at 5×10^6 cells/mL in doubly distilled water supplemented with 1× EQ four-element calibration beads (Fluidigm), and data was acquired on a CyTOF2 mass cytometer (Fluidigm). The data was then bead-normalized using MATLAB-based software [16]. The normalized data was

uploaded onto the Cytobank analysis platform[17] to perform initial gating and population identification using the indicated gating schemes. Data was represented as viSNE plots. viSNE schemes were created using the Cytobank online tool.

Flow cytometry. The gentleMACS™ Octo Dissociator (Miltenyi Biotec, Bergisch Gladbach, Germany) was used to dissociate the tumor tissue at 37 °C for 35 min. Samples were filtered through a 40-μm cell strainer to obtain a single-cell suspension. Cells (1 million/sample) were stained following blocking with anti-CD16/32 (anti-FcγIII/II receptor, clone 2.4G2, 20 min) with the primary antibodies for 20–30 min on ice. DAPI/Aqua dead cells marker was used to gate the dead cells. Details of the fluorescent-labeled antibodies are listed in Supplementary Table S9. Samples were analyzed in a Gallios flow cytometer or Cytoflex flow cytometer. Data were analyzed using FlowJo software or the Cytobank online tool**.**

MHC class-1 staining on tumor cells

4NQO-T and 4NQO-L cells were treated with trametinib (20nM), IFNγ (10ng/mL) and a combination of both. Cells were harvested after 24h and analyzed for MHC-I expression on the tumor cells using cytoflex flow-cytometer.

In vitro T-cell proliferation assay

Lymphocytes were enriched from the tumors treated as indicated and cultured with 20ng/mL IL2 (Miltenyi Biotec 130-120-662) for 5-6 days in αCD3 pre-coated 96 well plates**.** Proliferation of $CD8^+$ T cells was determined using the CFSE Cell Division Tracker Kit (Bio legend 423801) according to the manufacturer's protocols.

Intracellular IFNγ **staining.** Lymphocytes were enriched from tumors treated with vehicle or trametinib for 5 days and activated with 25 ng/mL PMA [Santa Cruz Biotechnology (sc-3576)] and 1 µM ionomycin [Santa Cruz Biotechnology (sc-3576)] for 5 h. After activation, cells were stained with the appropriate surface markers [dead cell marker-Zombie Aqua™ Fixable Viability Kit (Biolegend)] and CD8 (APC conjugated) and then fixed using 4% PFA for 20 min

Prasad M, *et al. J Immunother Cancer* 2022; 10:e003917. doi: 10.1136/jitc-2021-003917

at room temperature. Then cells were permeabilized with permeabilization buffer (1X) (eBioscience -00-8333-56) and stained with IFNγ-PE-cy7 for 20 min. Cells were then washed, and flow cytometry analysis was performed using Gallios flow cytometer. Data were analyzed using FlowJo software or Cytobank online tool.

Co-culture of CD8+ T cells and MDSCs, and T-cell proliferation. MDSCs, were sorted from tumors using a FACSAria II flow cytometer based on the expression of CD11b$^+$, F4/80$^+$, CD11c$^+$, MHC11$^+$ CSFR$^+$, CD11b$^+$, F480$^-$, CD11c$^+$, and MHC11$^+$ CSFR$^+$ cells, Naive CD8$^+$ T cells were purified from naïve C57/BL6 mice by using the mouse CD8$^+$ T Cell Isolation Kit (Miltenyi, 130-104-075). MDSCs and CD8$^+$ T cells were co-cultured at a ratio of 1:10 for 96 h. T cells were activated with 25 ng/mL PMA and 1 µM ionomycin. Proliferation of CD8$^+$ T cells was determined using the CFSE Cell Division Tracker Kit (Biolegend-423801) according to the manufacturer's protocols.

Generation of CSF-1-overexpressing cell lines. A lentiviral vector for CSF-1 (pLV-EGFP: T2A: Puro-EF1A>mCSF-1 [NM_001113530.1] and an EGFP lentiviral control vector (pLV-EGFP/Puro-CMV>Stuffer300) were ordered from VectorBuilder. 4NQO-T and 4NQO-L cells were infected with lentiviruses encoding for murine CSF-1 and EGFP as control, and the infected cells were selected using puromycin (5µg/mL).

RNA sequencing. RNA sequencing was performed on the 4NQO-L, 4NQO-T, 4NQO-L-PE, and 4NQO-T-PE cells. According to the manufacturer's instructions, RNA was extracted using the RNeasy mini kit (Qiagen, 74104). RNA-seq libraries were prepared as described previously by Elkabets lab[10]. Briefly, RNA-seq libraries were prepared using the Tru Seq RNA Sample Preparation kit (Illumina, San Diego, CA, USA) according to the manufacturer's protocol. Total RNA, 1 mg, was fragmented, followed by reverse transcription and second-strand cDNA synthesis. The DS-cDNA was subjected to end repair, a base addition, adapter ligation, and PCR

Prasad M, *et al. J Immunother Cancer* 2022; 10:e003917. doi: 10.1136/jitc-2021-003917

amplification to create libraries. Sequencing was performed with a Nextseq 5000 system, using all four lanes.

RNA silencing experiment. For transient silencing of STAT3, cells were transfected using GenMute siRNA Transfection Reagent (SignaGen, SL100568) according to the manufacturer's protocol, with siRNAs nontargeting control sequence (IDT, 51-01-14-04) and STAT3 gene targeting sequences (IDT; mm.Ri.STAT3.13.1). Cells were harvested after 48 hours for western blotting and real-time PCR analysis.

Real-time quantitative PCR. Total RNA was isolated from 4NQO-L, 4NQO-T, 4NQO-L-PE, and 4NQO-T-PE cells treated with trametinib (20 nM) for different times, using ISOLATE II RNA Mini Kit (Bioline- BIO-52073) according to the manufacturer's protocol. RNA, 1 µg, was converted to cDNA using a qScript cDNA synthesis kit (Quanta Bioscience, 95047-100) according to the manufacturer's protocol. Real-time PCR was performed (Roche LightCycler® 480 II) using a prime time gene expression master mix (IDT, 1055770), with matching probes from IDT: CSF-1 gene mouse (Mm.PT.58.11661276) and GAPDH mouse (Mm.PT.39a.1).

CLB-IHN cohorts. The CLB-IHN cohort is derived from a previously published cohort of patients treated at CLB (Lyon France) for a histologically confirmed recurrent or metastatic head and neck SCC in clinical trials testing the efficacy of PD-1/PD-L1 antibodies alone or in combination with an anti-KIR or an anti-CTLA4 antibody between March 2014 and November 2018[18] (Supplementary Table S5). Targeted gene expression profiles (HTG EdgeSeq technology, Oncology Biomarker Panel [OBP]) were generated for pre-immunotherapy formalin-fixed, paraffin-embedded (FFPE) tumor samples. Briefly, the median age of the patients was 63 years (range 33-88). Most patients were male (81%), current/former smokers (87%), and alcohol drinkers (85%). HNC originated mainly in the oropharynx (39%) and oral cavity (33%) and best response on immune checkpoint immunotherapy were only 11% with partial response and complete response.

Statistical analysis. Each experiment was repeated 2 or 3 times, and representative data are shown. Statistical analysis was performed using GraphPad Prism 7 software, and results are presented as means ± SEM. IHC images were analyzed by Histoquant software (3DHISTECH), and differences between the two groups were analyzed using an independent t-test, whereas for the analysis of more than two groups a one-way ANOVA test was used. Correlation analysis was conducted using Spearman's rho test. Overall survival (OS) was defined by the time in months from tumor biopsy to death or loss to follow-up. In the CLB-IHN cohort, survival distributions were estimated using the Kaplan–Meier method and compared with the log-rank test between patients with a high vs. a low value of the CD8A/CSF1 ratio (binarization at the median). Survival analyses were performed using R 4.0.0 and the survival_3.1-12, survminer_0.4.7, and ggplot2 packages. For all the experiments, P values of 0.05, 0.01, 0.001 or 0.0001 were considered statistically significant, as designated by *, **, ***, ****, respectively.

REFERENCES

1 Stransky N, Egloff AM, Tward AD, *et al.* The mutational landscape of head and neck squamous cell carcinoma. *Science (80-)* 2011;**333**:1157–60. doi:10.1126/science.1208130

2 Morris LGT, Chandramohan R, West L, *et al.* The molecular landscape of recurrent and metastatic head and neck cancers insights from a precision oncology sequencing platform. *JAMA Oncol* 2017;**3**:244–55. doi:10.1001/jamaoncol.2016.1790

3 Sweeney SM, Cerami E, Baras A, *et al.* AACR project genie: Powering precision medicine through an international consortium. *Cancer Discov* 2017;**7**:818–31. doi:10.1158/2159-8290.CD-17-0151

4 Agrawal N, Frederick MJ, Pickering CR, *et al.* Exome sequencing of head and neck squamous cell carcinoma reveals inactivating mutations in NOTCH1. *Science* 2011;**333**:1154–7.

doi:10.1126/science.1206923

5 Metsalu T, Vilo J. ClustVis: a web tool for visualizing clustering of multivariate data using Principal Component Analysis and heatmap. *Nucleic Acids Res* 2015;**43**:W566. doi:10.1093/NAR/GKV468

6 Schubert M, Klinger B, Klünemann M, *et al.* Perturbation-response genes reveal signaling footprints in cancer gene expression. *Nat Commun* 2018;**9**. doi:10.1038/S41467-017-02391-6

7 A M, M P, J M, *et al.* EGFR and PI3K Pathway Activities Might Guide Drug Repurposing in HPV-Negative Head and Neck Cancers. *Front Oncol* 2021;**11**:678966–678966. doi:10.3389/FONC.2021.678966

8 Liberzon A, Birger C, Thorvaldsdóttir H, *et al.* The Molecular Signatures Database Hallmark Gene Set Collection. *Cell Syst* 2015;**1**:417–25. doi:10.1016/J.CELS.2015.12.004

9 Subramanian A, Tamayo P, Mootha VK, *et al.* From the Cover: Gene set enrichment analysis: A knowledge-based approach for interpreting genome-wide expression profiles. *Proc Natl Acad Sci U S A* 2005;**102**:15545. doi:10.1073/PNAS.0506580102

10 Yegodayev KM, Novoplansky O, Golden A, *et al.* TGF-beta-activated cancer-associated fibroblasts limit cetuximab efficacy in preclinical models of head and neck cancer. *Cancers (Basel)* 2020;**12**. doi:10.3390/cancers12020339

11 Remer E, Badarni M, Hikri E, *et al.* CDK 4/6 Inhibition Overcomes Acquired and Inherent Resistance to PI3Kα Inhibition in Pre-Clinical Models of Head and Neck Squamous Cell Carcinoma. *J Clin Med* 2020;**9**:3214. doi:10.3390/jcm9103214

12 Badarni M, Prasad M, Balaban N, *et al.* Repression of AXL expression by AP-1/JNK blockage overcomes resistance to PI3Ka therapy. *JCI Insight* 2019;**4**. doi:10.1172/JCI.INSIGHT.125341

13 Prasad M, Jagadeeshan S, Scaltriti M, *et al.* In vitro establishment of a genetically engineered murine head and neck cancer cell line using an adeno-associated Virus-Cas9 system. *J Vis Exp*

2019;**2020**:e60410. doi:10.3791/60410

14 Hoover AC, Spanos WC, Harris GF, *et al.* The role of human papillomavirus 16 E6 in anchorage-independent and invasive growth of mouse tonsil epithelium. *Arch Otolaryngol Head Neck Surg* 2007;**133**:495–502. doi:10.1001/ARCHOTOL.133.5.495

15 Vermeer DW, Coppock JD, Zeng E, *et al.* Metastatic model of HPV+ oropharyngeal squamous cell carcinoma demonstrates heterogeneity in tumor metastasis. *Oncotarget* 2016;**7**:24194–207. doi:10.18632/ONCOTARGET.8254

16 Finck R, Simonds EF, Jager A, *et al.* Normalization of mass cytometry data with bead standards. *Cytom Part A* 2013;**83 A**:483–94. doi:10.1002/cyto.a.22271

17 Kotecha N, Krutzik PO, Irish JM. Web-based analysis and publication of flow cytometry experiments. Curr. Protoc. Cytom. 2010;**Chapter 10**. doi:10.1002/0471142956.cy1017s53

18 Karabajakian A, Garrivier T, Crozes C, *et al.* Hyperprogression and impact of tumor growth kinetics after PD1/PDL1 inhibition in head and neck squamous cell carcinoma. *Oncotarget* 2020;**11**:1618–28. doi:10.18632/oncotarget.27563
