# Supplementary material for: MEK1/2 inhibition transiently alters the tumor immune microenvironment to enhance immunotherapy efficacy against head and neck cancer
Source: J Immunother Cancer. 2022 Mar 15;10(3):e003917. doi: 10.1136/jitc-2021-003917 (PMC8928405; doi:10.1136/jitc-2021-003917)
Supplement: Supplementary data [file jitc-2021-003917supp002.pdf]

Figure S1

A

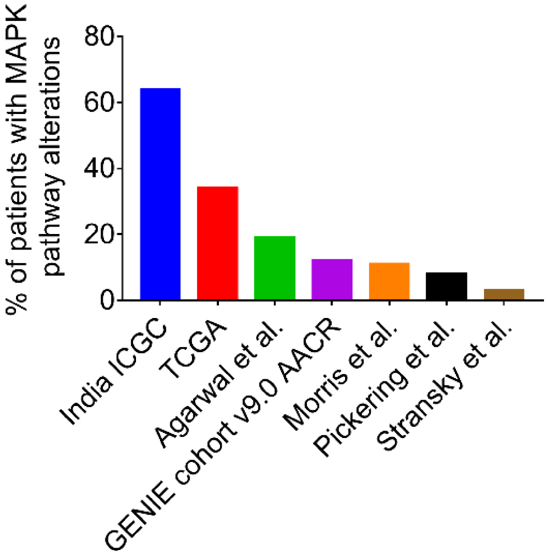

B

Broad Science 2011, Johns Hopkins Science 2011, TCGA Firehouse Legacy, MD Anderson, Cancer Discovery 2013

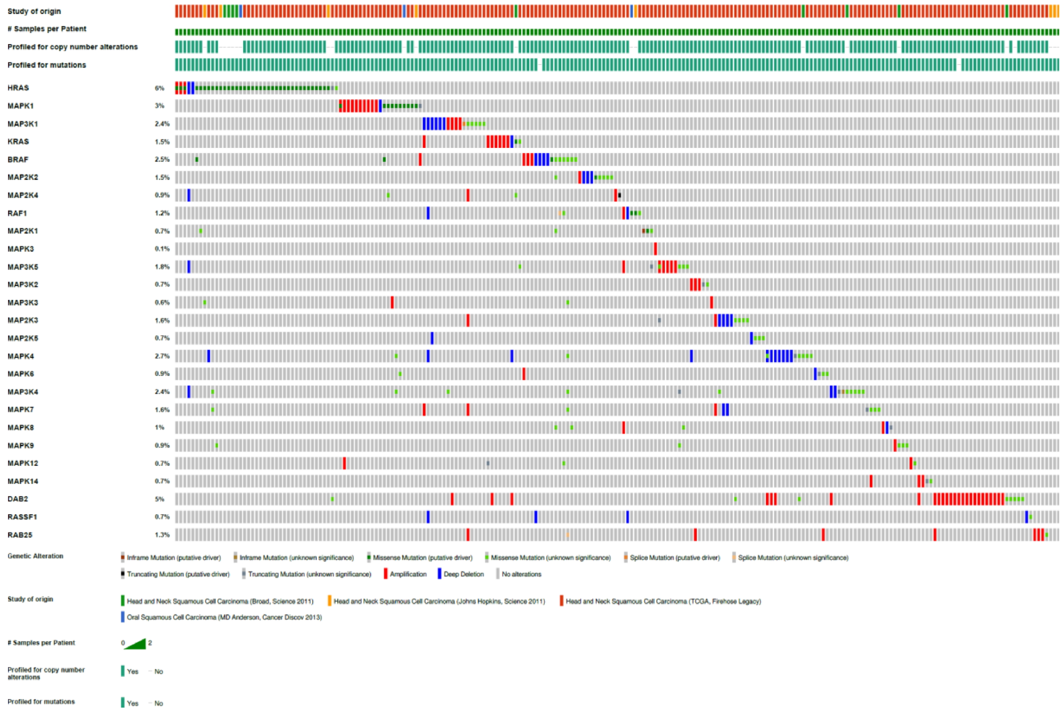

Figure S1

C

GENIE v9.1

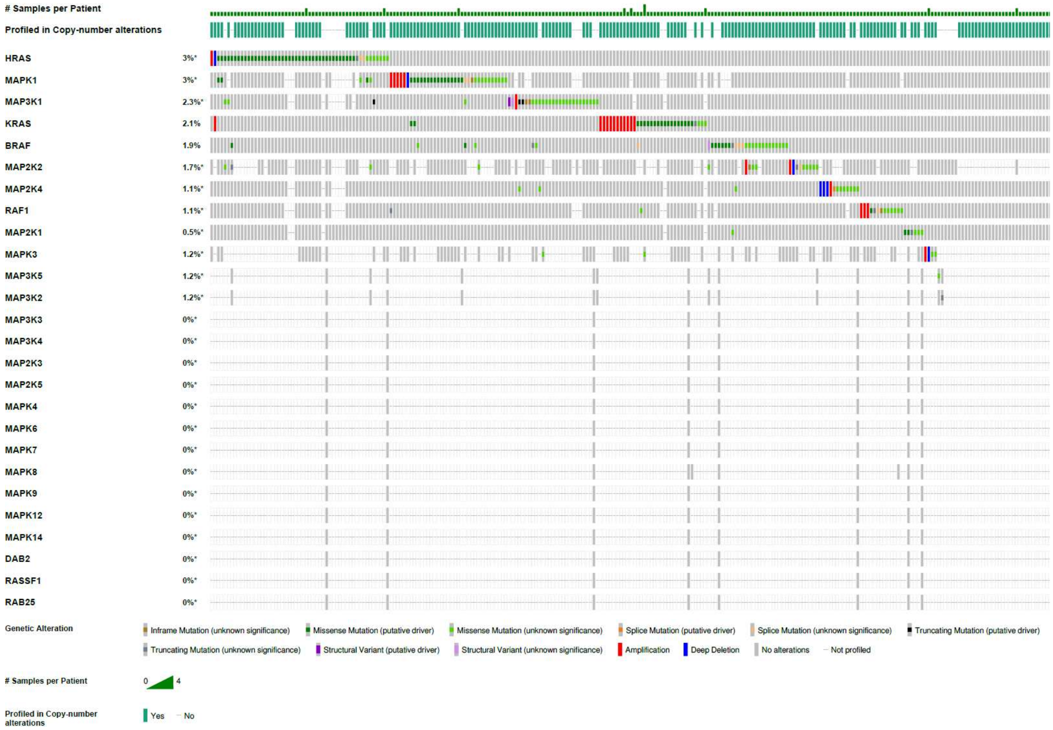

Figure S1

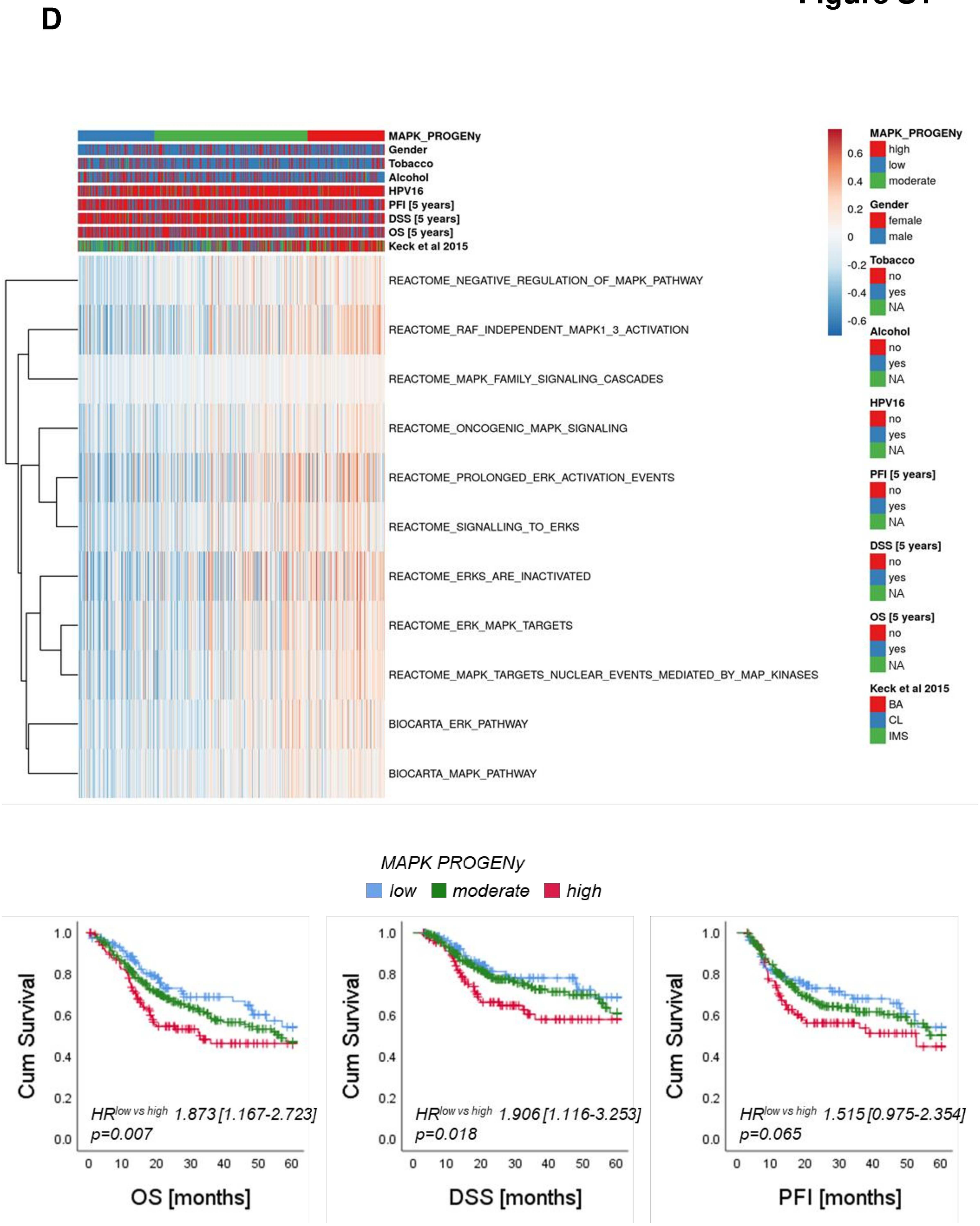
D
MAPK_PROGENy
Gender
Tobacco
Alcohol
HPV16
PFI [5 years]
DSS [5 years]
OS [5 years]
Keck et al 2015
REACTOME_NEGATIVE_REGULATION_OF_MAPK_PATHWAY
REACTOME_RAF_INDEPENDENT_MAPK1_3_ACTIVATION
REACTOME_MAPK_FAMILY_SIGNALING_CASCADES
REACTOME_ONCOGENIC_MAPK_SIGNALING
REACTOME_PROLONGED_ERK_ACTIVATION_EVENTS
REACTOME_SIGNALLING_TO_ERKS
REACTOME_ERKS_ARE_INACTIVATED
REACTOME_ERK_MAPK_TARGETS
REACTOME_MAPK_TARGETS_NUCLEAR_EVENTS_MEDIATED_BY_MAP_KINASES
BIOCARTA_ERK_PATHWAY
BIOCARTA_MAPK_PATHWAY
0.6
0.4
0.2
0
-0.2
-0.4
-0.6
MAPK_PROGENy high low moderate
Gender female male
Tobacco no yes NA
Alcohol no yes NA
HPV16 no yes NA
PFI [5 years] no yes NA
DSS [5 years] no yes NA
OS [5 years] no yes NA
Keck et al 2015 BA CL IMS
MAPK PROGENy
low moderate high
Cum Survival
HR low vs high 1.873 [1.167-2.723]
p=0.007
OS [months]
HR low vs high 1.906 [1.116-3.253]
p=0.018
DSS [months]
HR low vs high 1.515 [0.975-2.354]
p=0.065
PFI [months]

Figure S1

E

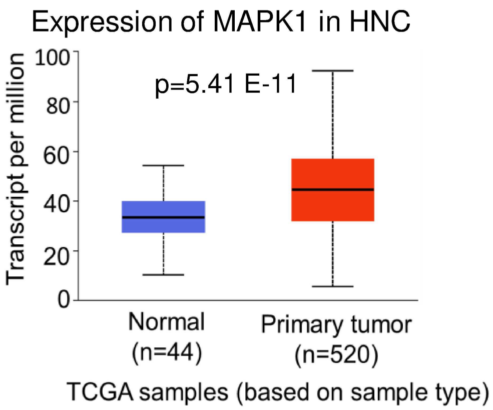

F

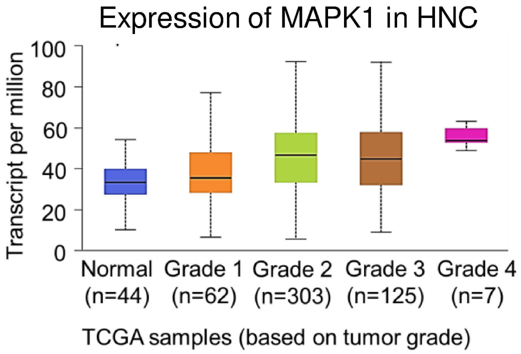

Comparison	Statistical significance
Normal-vs-Grade 1	2.026100E-02
Normal-vs-Grade 2	4.42257341859431E-12
Normal-vs-Grade 3	9.2663000050841E-08
Normal-vs-Grade 4	9.918200E-03
Grade 1-vs-Grade 2	4.198400E-03
Grade 1-vs-Grade 4	2.234100E-02

G

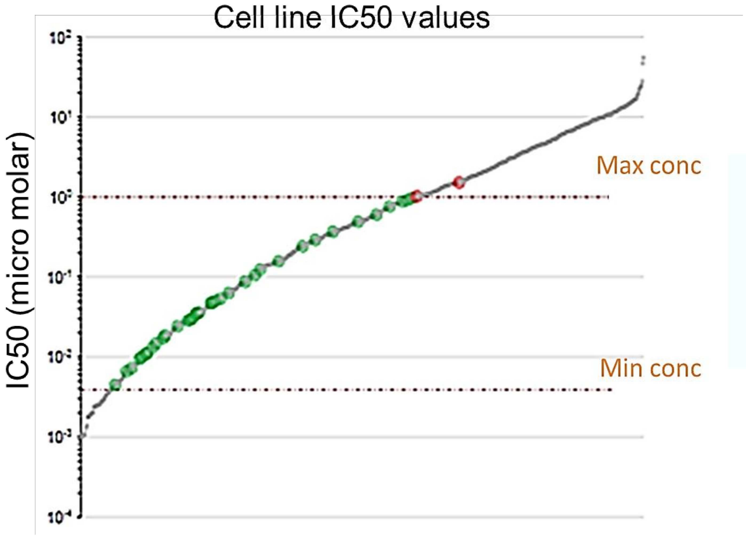

Number of cell lines screened	38
Maximum IC50 (uM)	1.53
Geometric mean (uM)	0.0668
Minimum IC50 (uM)	0.00445
Min screening concentration (uM)	0.00391
Max screening concentration (uM)	1.00

Figure S1

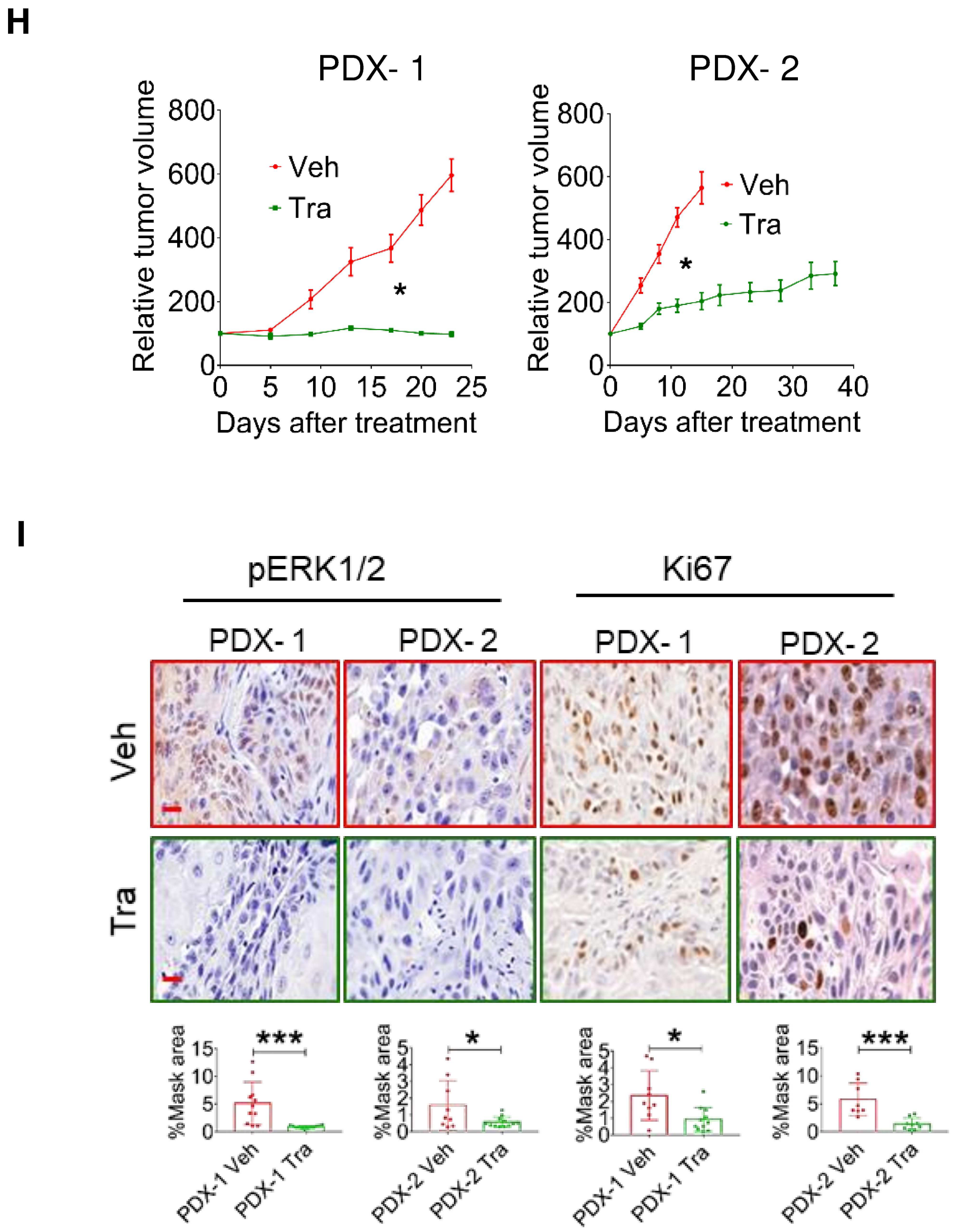
H
PDX- 1
PDX- 2
Relative tumor volume
Days after treatment
Veh
Tra
*
0
200
400
600
800
0 5 10 15 20 25
0 10 20 30 40
I
pERK1/2
Ki67
PDX- 1
PDX- 2
Veh
Tra
%Mask area
***
*
PDX-1 Veh
PDX-1 Tra
PDX-2 Veh
PDX-2 Tra

Figure S1

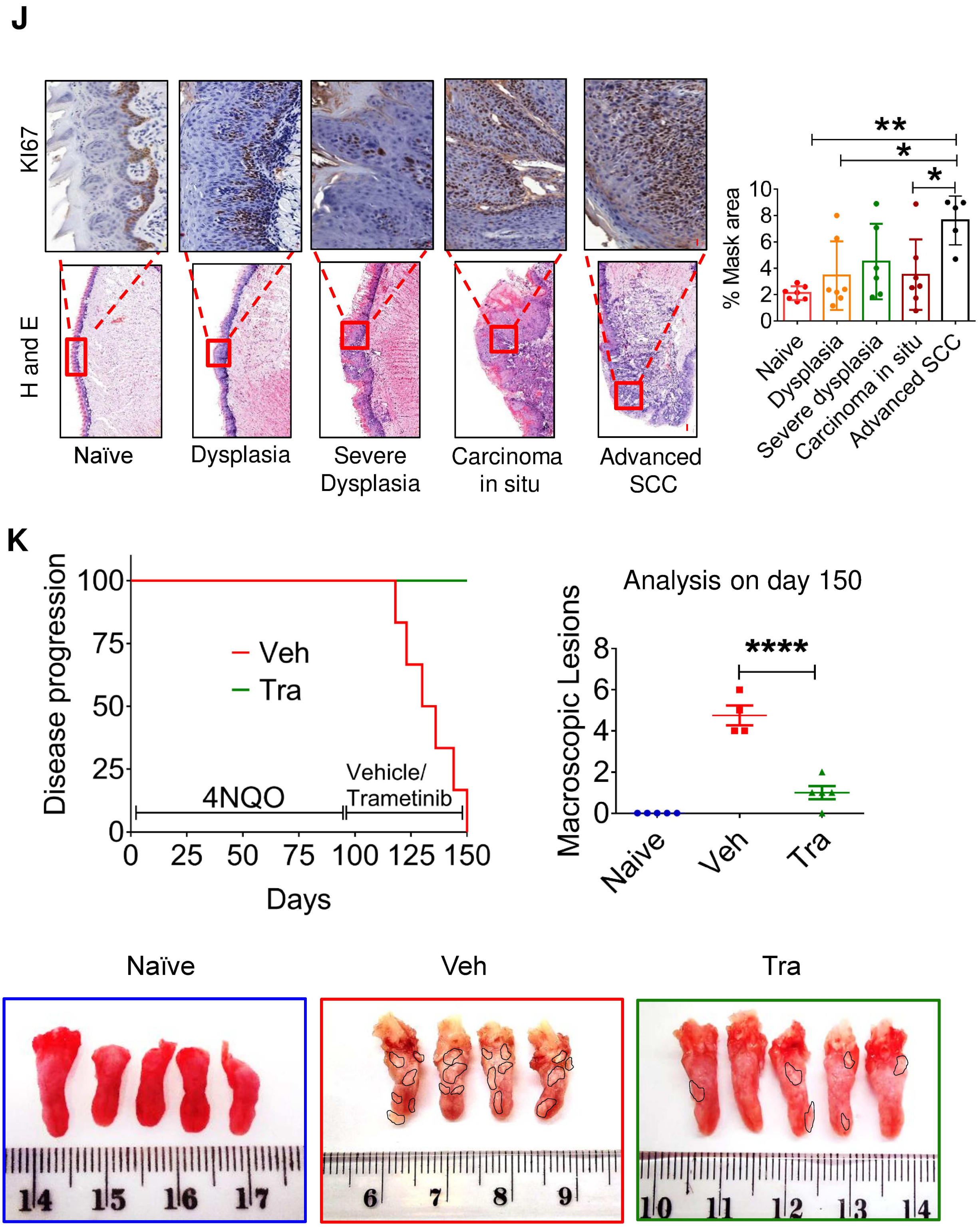
J
KI67
H and E
Naïve
Dysplasia
Severe Dysplasia
Carcinoma in situ
Advanced SCC
% Mask area
Naive
Dysplasia
Severe dysplasia
Carcinoma in situ
Advanced SCC
**
*
*
K
Disease progression
Days
Veh
Tra
4NQO
Vehicle/ Trametinib
Analysis on day 150
Macroscopic Lesions
****
Naive
Veh
Tra
Naïve
Veh
Tra

Supplementary Figure S1. (A) Genomics analysis of multiple HNC cohorts showing the percentage of patients with alterations in genes of the MAPK pathway. (**B)** MAPK mutated genes in HNC patients – joint analysis of five cohorts of whole-exome sequencing. (**C)** MAPK mutated genes in HNC patients – analysis of the GENIE cohort. (**D)** Top – Heatmap illustrates GSVA score distribution for selected MAPK-related gene sets among tumors of TCGA-HNSC ranked according to MAPK activity inferred with the PROGENy algorithm. NA, not available. Bottom – Kaplan-Meier plots for five-year overall survival (OS; left), disease specific survival (DSS; middle) and progression-free intervals (PFI; right) for tumors with low, moderate and high MAPK pathway activity according to the PROGENy algorithm. (**E)** MAPK1 expression in normal tissue and in primary HNC tumors (TCGA database). (**F)** MAPK1 expression in different pathological grades of HNC (TCGA database); the table on the right presents the p values. (**G)** Analysis of the Cancer Cell Line Encyclopedia (CCLE) and Sanger cell line datasets showing the sensitivity of 38 cell lines to the MEK1/2 inhibitor, trametinib. (**H)** Relative tumor growth of two HNC-PDXs after treatment with trametinib. Values in the graphs are mean tumor volumes ± SEM. **(I)** Top – IHC staining of pERK1/2 and Ki67 in tissues of two HNC-PDXs after treatment with trametinib. Bottom – quantification of staining performed using 3DHISTECH software HistoQuant (n > 10) (scale bar: 10 µm) **(J)** IHC images (top) of Ki67 staining and H&E images (bottom) showing various stages of oral (tongue) carcinogenesis induced by 4NQO in C57BL/6J mice. The percentage of mask area and statistics are also shown. Scale bars: 20 µm (top); 200 µm (bottom). (**K)** Top panel – disease progression of 4NQO-induced oral cancer (left) and quantification of macroscopic lesions (right). Bottom panel – Photographs showing the macroscopic lesions in the tongues of naïve, vehicle-exposed, and trametinib-treated mice after 150 days of the experiment. For statistics, an unpaired 2-sided t-test or one-way ANOVA was performed. *p < 0.05; **p < 0.01; ***p < 0.001, ****p < 0.0001 were considered statistically significant. Tra - trametinib, Veh - vehicle.

Figure S2

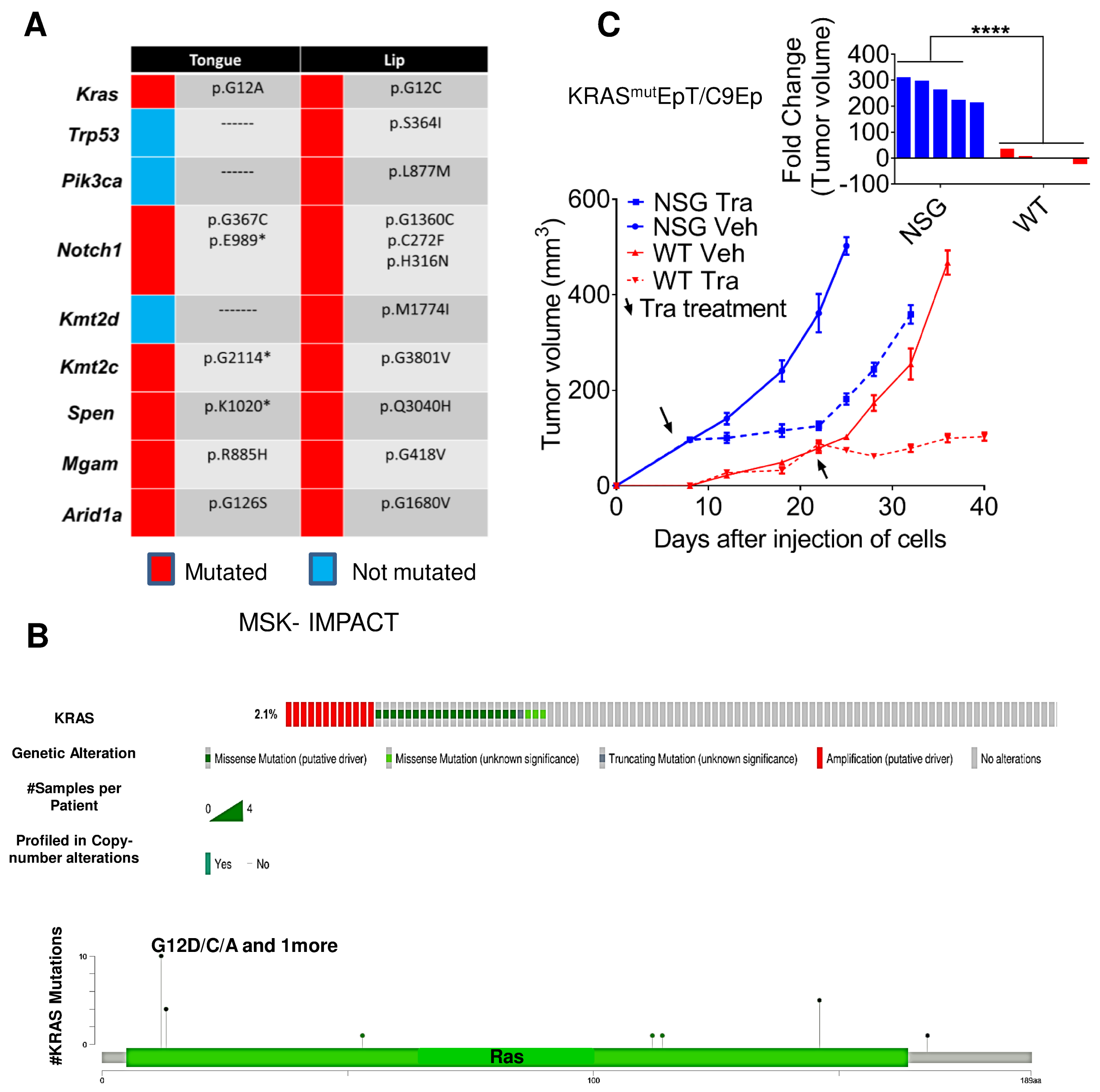
A
Tongue
Lip
Kras
p.G12A
p.G12C
Trp53
------
p.S364I
Pik3ca
------
p.L877M
Notch1
p.G367C
p.E989*
p.G1360C
p.C272F
p.H316N
Kmt2d
----
p.M1774I
Kmt2c
p.G2114*
p.G3801V
Spen
p.K1020*
p.Q3040H
Mgam
p.R885H
p.G418V
Arid1a
p.G126S
p.G1680V
Mutated
Not mutated
B
MSK- IMPACT
KRAS
2.1%
Genetic Alteration
Missense Mutation (putative driver)
Missense Mutation (unknown significance)
Truncating Mutation (unknown significance)
Amplification (putative driver)
No alterations
#Samples per Patient
0
4
Profiled in Copy-number alterations
Yes
No
#KRAS Mutations
G12D/C/A and 1more
10
0
Ras
0
100
189aa
C
KRASmutEpT/C9Ep
Fold Change (Tumor volume)
****
400
300
200
100
0
-100
NSG
WT
Tumor volume (mm3)
600
400
200
0
NSG Tra
NSG Veh
WT Veh
WT Tra
Tra treatment
0
10
20
30
40
Days after injection of cells

Figure S2

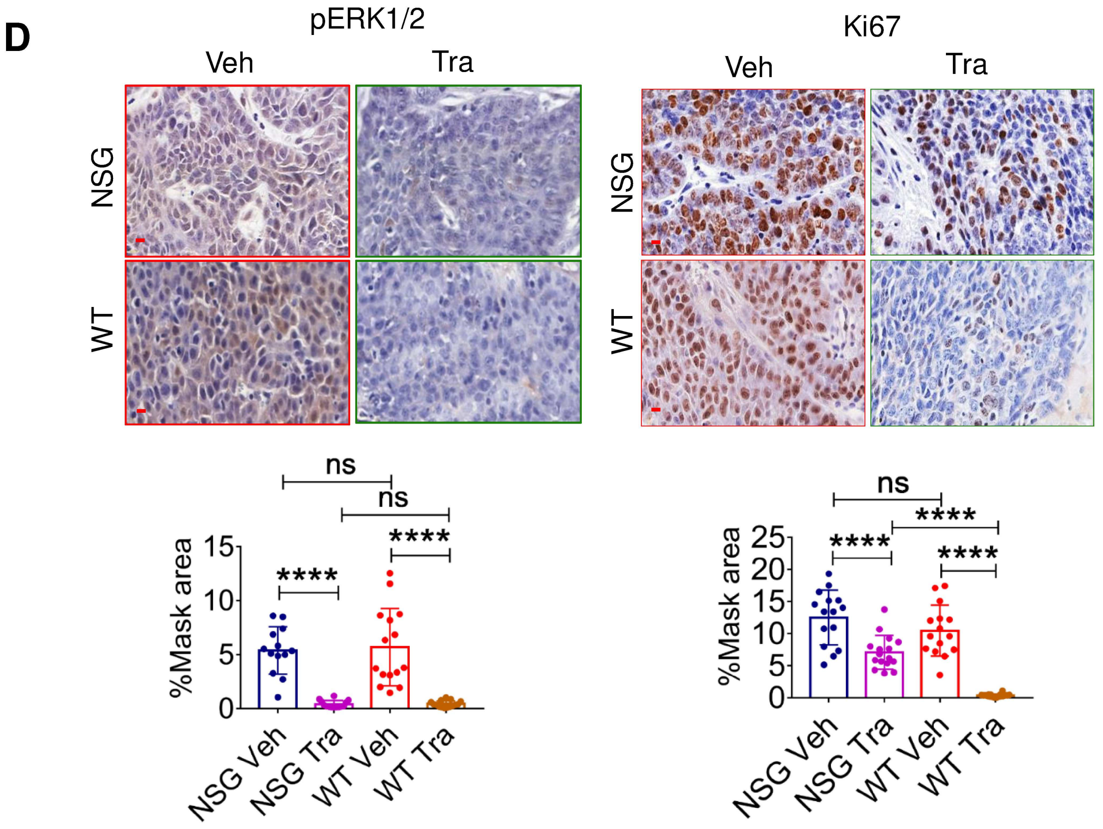
D
pERK1/2
Veh
Tra
NSG
WT
Ki67
Veh
Tra
NSG
WT
ns
ns
****
****
%Mask area
15
10
5
0
NSG Veh
NSG Tra
WT Veh
WT Tra
ns
****
****
****
%Mask area
25
20
15
10
5
0
NSG Veh
NSG Tra
WT Veh
WT Tra

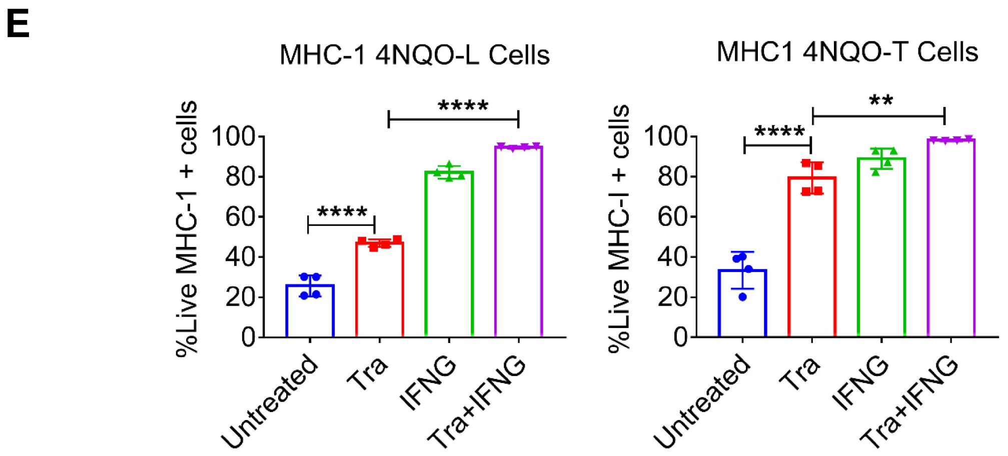
E
MHC-1 4NQO-L Cells
****
****
%Live MHC-1 + cells
100
80
60
40
20
0
Untreated
Tra
IFNG
Tra+IFNG
MHC1 4NQO-T Cells
**
****
%Live MHC-I + cells
100
80
60
40
20
0
Untreated
Tra
IFNG
Tra+IFNG

Figure S2

F

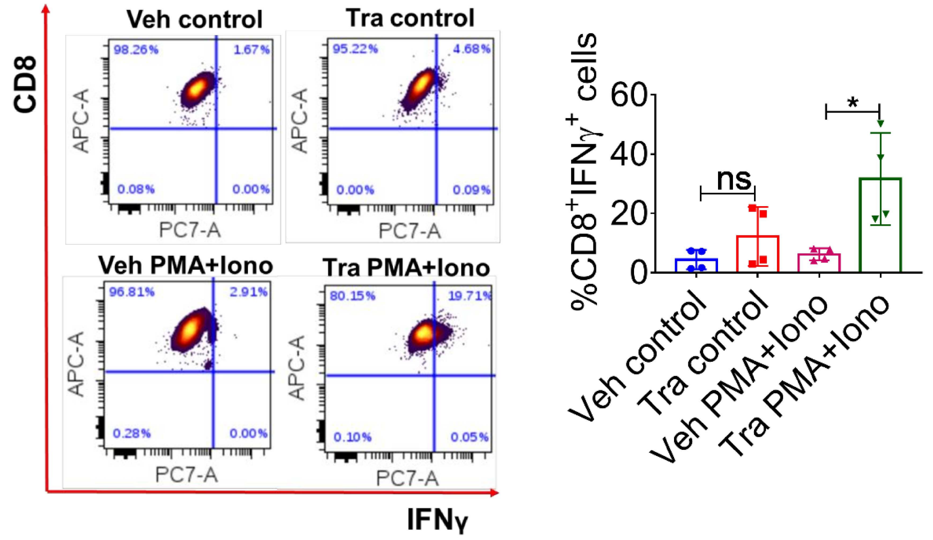

G

FMO controls

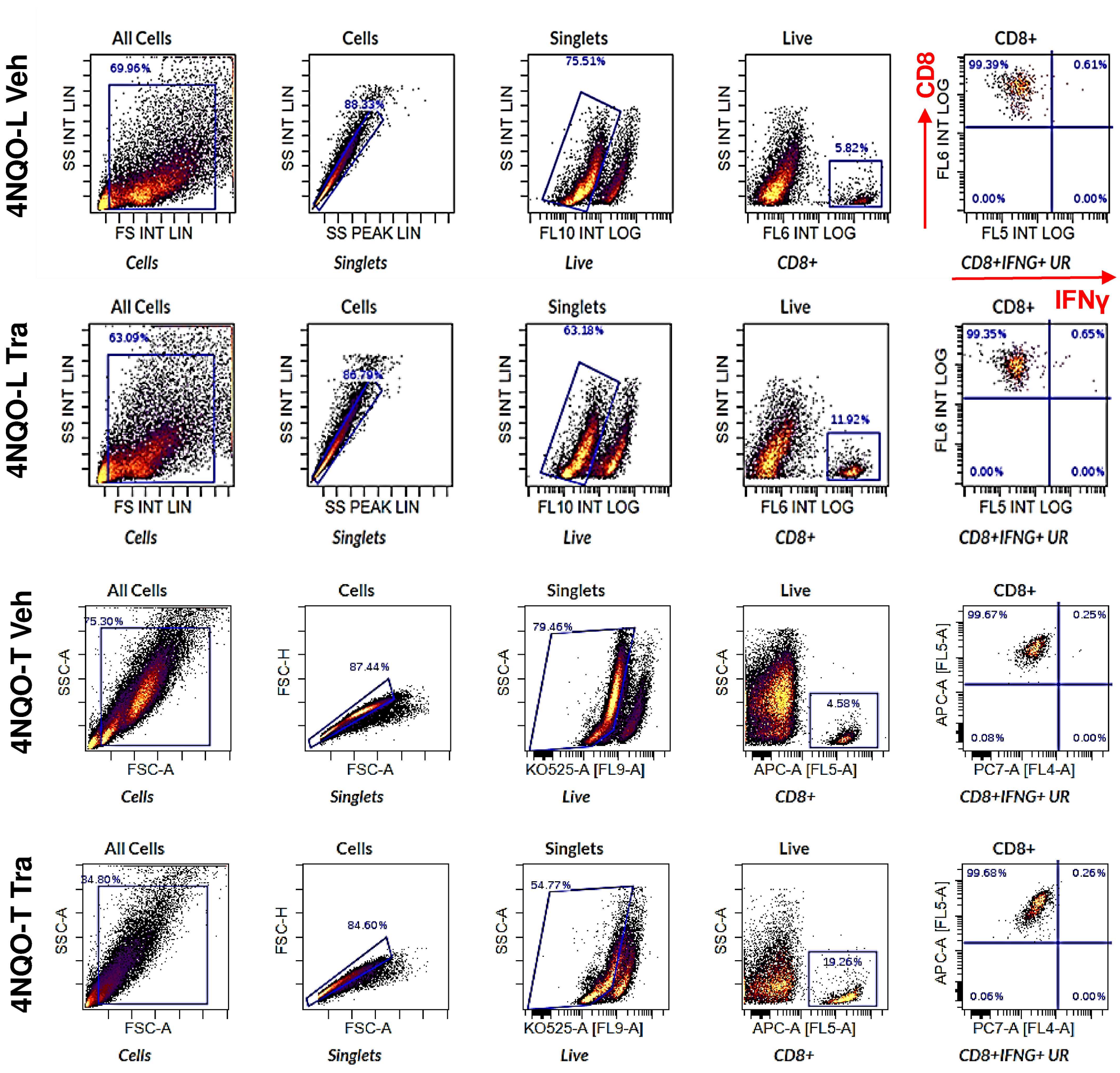

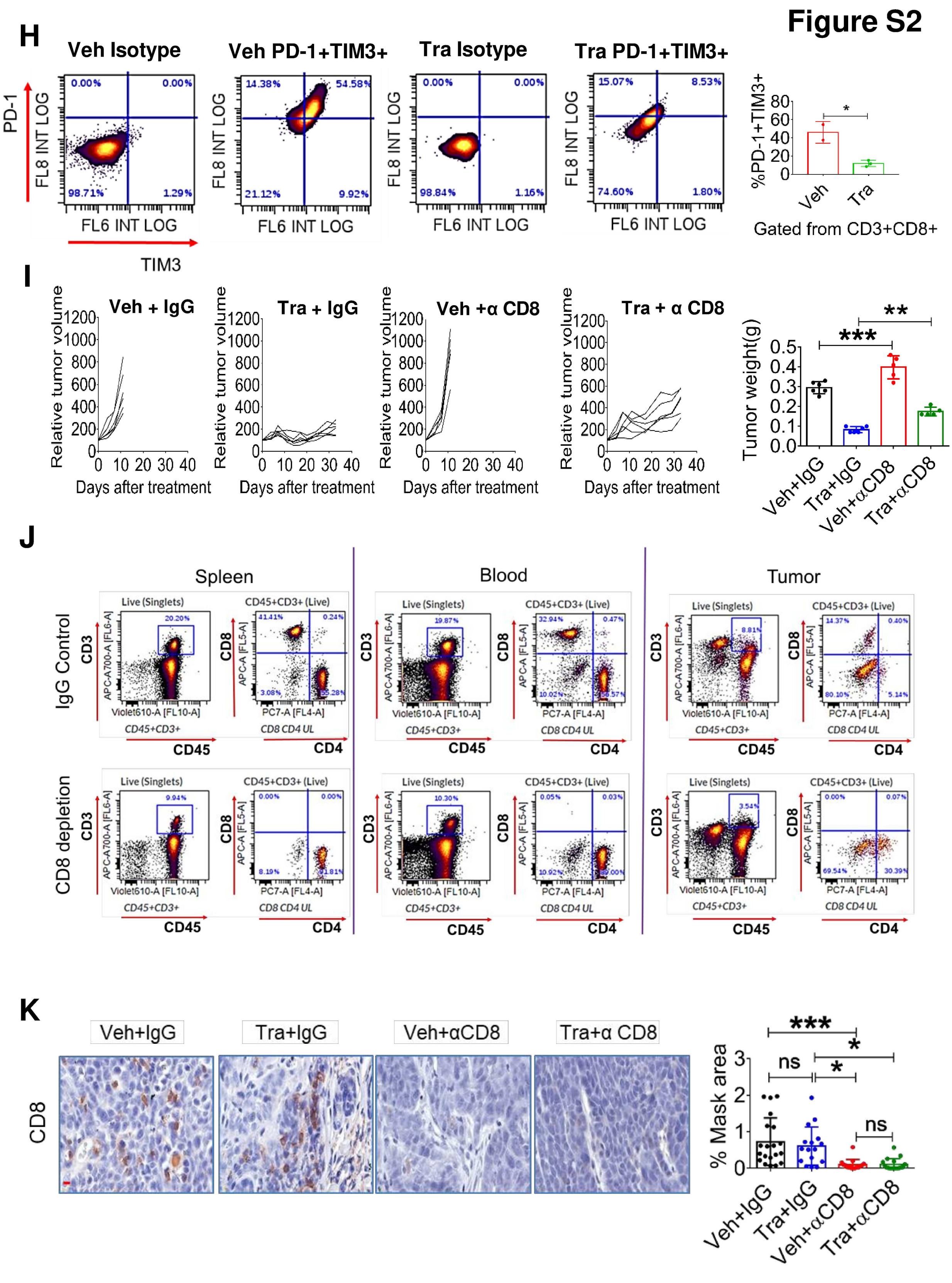
Figure S2
H
Veh Isotype
Veh PD-1+TIM3+
Tra Isotype
Tra PD-1+TIM3+
PD-1
FL8 INT LOG
FL6 INT LOG
TIM3
%PD-1+TIM3+
Veh
Tra
Gated from CD3+CD8+
I
Veh + IgG
Tra + IgG
Veh +α CD8
Tra + α CD8
Relative tumor volume
Days after treatment
Tumor weight(g)
Veh+IgG
Tra+IgG
Veh+αCD8
Tra+αCD8
J
Spleen
Blood
Tumor
IgG Control
CD8 depletion
Live (Singlets)
CD45+CD3+ (Live)
CD3
CD8
CD45
CD4
K
Veh+IgG
Tra+IgG
Veh+αCD8
Tra+α CD8
CD8
% Mask area
ns

Supplementary Figure S2. (A) Key mutations in 4NQO-T and 4NQO-L cell lines found in MSK-IMPACT genome sequencing. (**B)** Hot spot mutations of KRAS in HNC, extracted from cBioportal, GENIE cohort. **(C)** Growth curve and statistics of $KRAS^{mut}$EpT/C9Ep tumors in NSG and WT mice treated with vehicle or trametinib (**D)** IHC staining of pERK1/2 (top left) and Ki67 (top right) in NSG and WT mice (4NQO-L). Quantification is shown in the bottom panel (scale bars: 20 µm; inset 10 µm). **(E)** MHC class-I expression on the surface of 4NQO-T and 4NQO-L cell lines treated with trametinib, IFNγ and trametinib + IFNγ (**F)** Intracellular staining of IFNγ in $CD8^+$ T cells isolated from the 4NQO-T vehicle-treated or trametinib SE-treated (5 days) mice. Graph on right showing the percentage of $CD8^+IFN\gamma^+$ with or without activation with phorbol 12-myristate 13-acetate (PMA) and ionomycin (Iono). Data from two independent experiments are shown. **G)** Representative images of gating scheme of FMO controls of IFNγ staining of $CD8^+$ T cells from veh and tra treated 4NQO-L and 4NQO-T tumors **(H)** PD-1 and TIM3 levels in $CD8^+$ T cells in 4NQO-L tumors treated with vehicle or a short exposure with trametinib with isotype controls. **(I)** Left– Tumor growth curves for four groups of mice: vehicle + IgG, trametinib + IgG, vehicle + anti-CD8 (αCD8), and trametinib + αCD8. Right – tumor weight (g) of 4NQO-L tumors in WT mice treated with vehicle or trametinib with and without depletion of $CD8^+$ T cells. (**J)** Depletion efficiency of $CD8^+$ T cells by flow cytometry analysis of spleen, blood and tumors of mice treated for 5 days with IgG or anti-CD8. **(K)** IHC staining and quantification of $CD8^+$ T cell in tissues of CD8 depletion experiment (scale bar: 20 µm). For statistics, an unpaired 2-sided t-test or one-way ANOVA was performed. $*p < 0.05$; $**p < 0.01$; $***p < 0.001$, $****p < 0.0001$ were considered statistically significant. Tra - trametinib, Veh - vehicle.

Figure S3

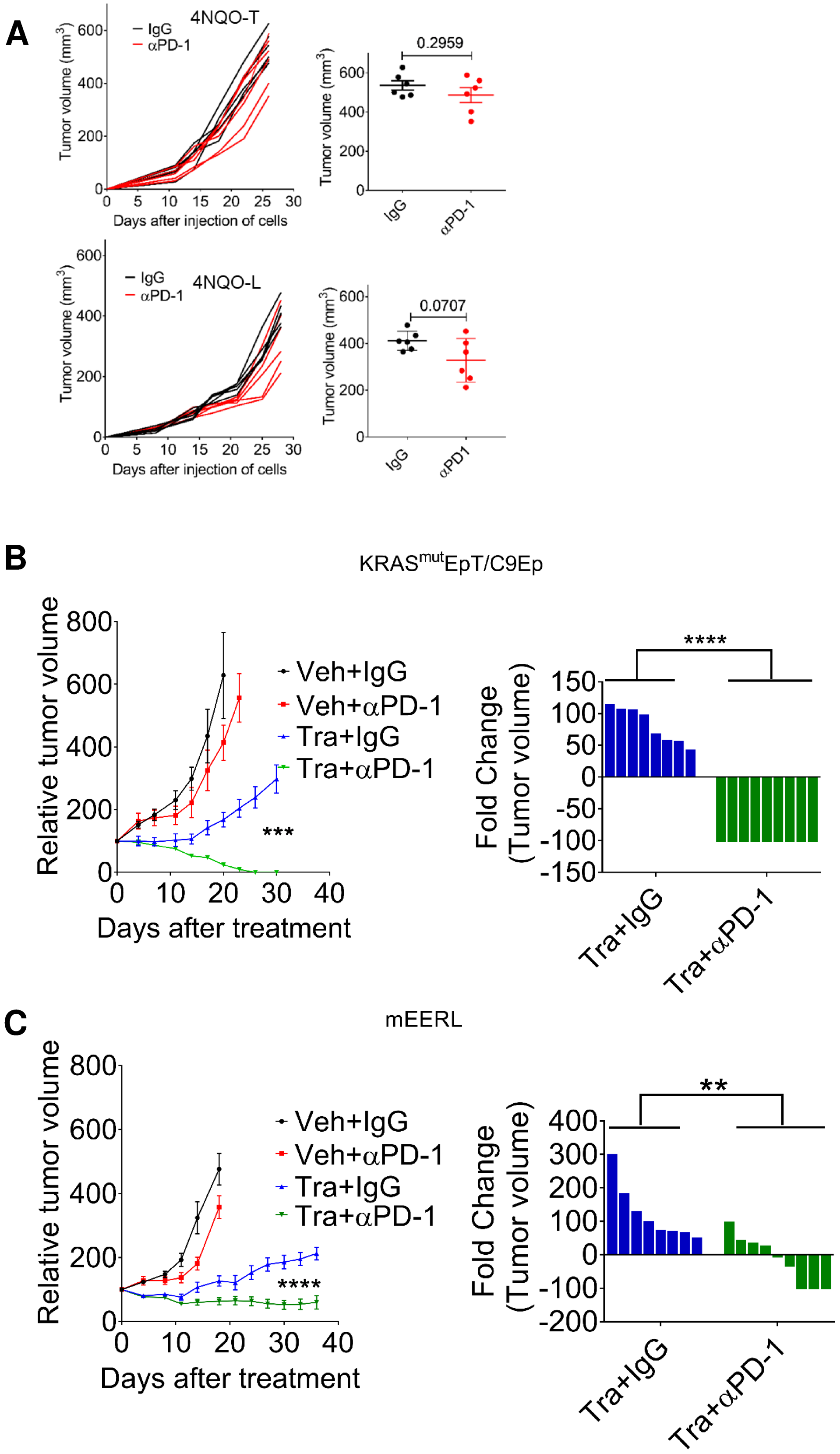
A
4NQO-T
IgG
αPD-1
Tumor volume (mm3)
Days after injection of cells
0.2959
4NQO-L
0.0707
αPD1
B
KRASmutEpT/C9Ep
Relative tumor volume
Days after treatment
Veh+IgG
Veh+αPD-1
Tra+IgG
Tra+αPD-1
***
****
Fold Change (Tumor volume)
C
mEERL
**

Figure S3

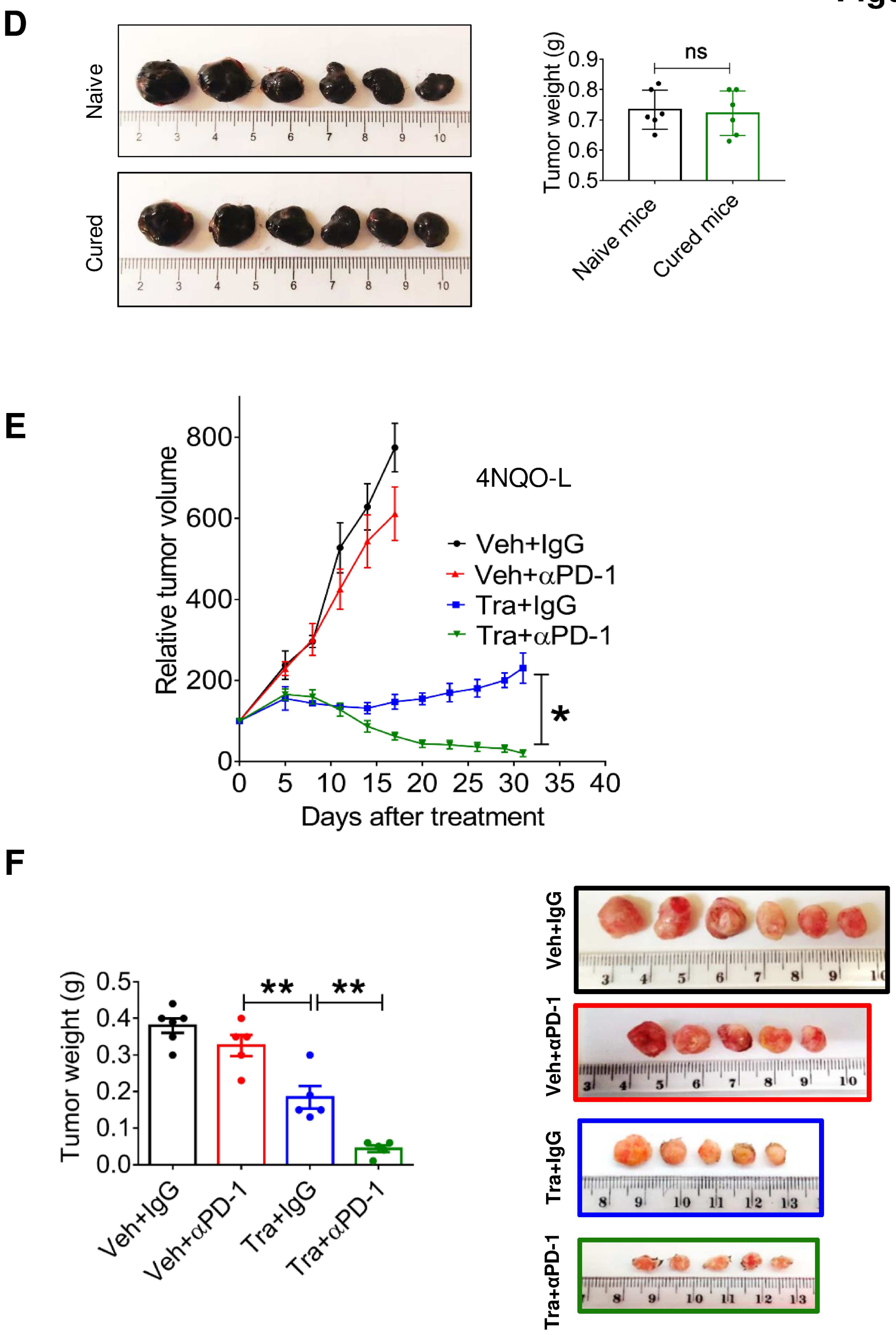
D
Naive
Cured
Tumor weight (g)
ns
Naive mice
Cured mice
E
4NQO-L
Relative tumor volume
Days after treatment
Veh+IgG
Veh+αPD-1
Tra+IgG
Tra+αPD-1
*
F
Tumor weight (g)
**
**
Veh+IgG
Veh+αPD-1
Tra+IgG
Tra+αPD-1

Supplementary Figure S3. (A) Tumor growth curves and volumes at the endpoint of the experiment for 4NQO-L and 4NQO-T tumors in WT mice treated with αPD-1 or IgG. (**B)** Relative tumor volumes of $KRAS^{mut}$EpT/C9Ep and (C) mEERL tumors in WT mice treated with αPD-1, trametinib or the combination of αPD-1 and trametinib. Left- Fold change of tumor volumes of $KRAS^{mut}$EpT/C9Ep and mEERL tumors treated with trametinib/IgG Vs trametinib/αPD-1. (D) B16 tumors in naïve and cured mice and tumor weights in grams (bar diagram). (**E)** Relative volumes of 4NQO-L tumors in WT mice treated as indicated. (**F)** Weights of 4NQO-L tumors and images of the tumors at the end of the experiment (day 31). For statistics, an unpaired 2-sided *t*-test or one-way ANOVA was performed. *p < 0.05, **p < 0.01, ***p < 0.001, and ****p< 0.0001 were considered statistically significant. ns - not significant. Tra - trametinib, Veh - vehicle.

Figure S4

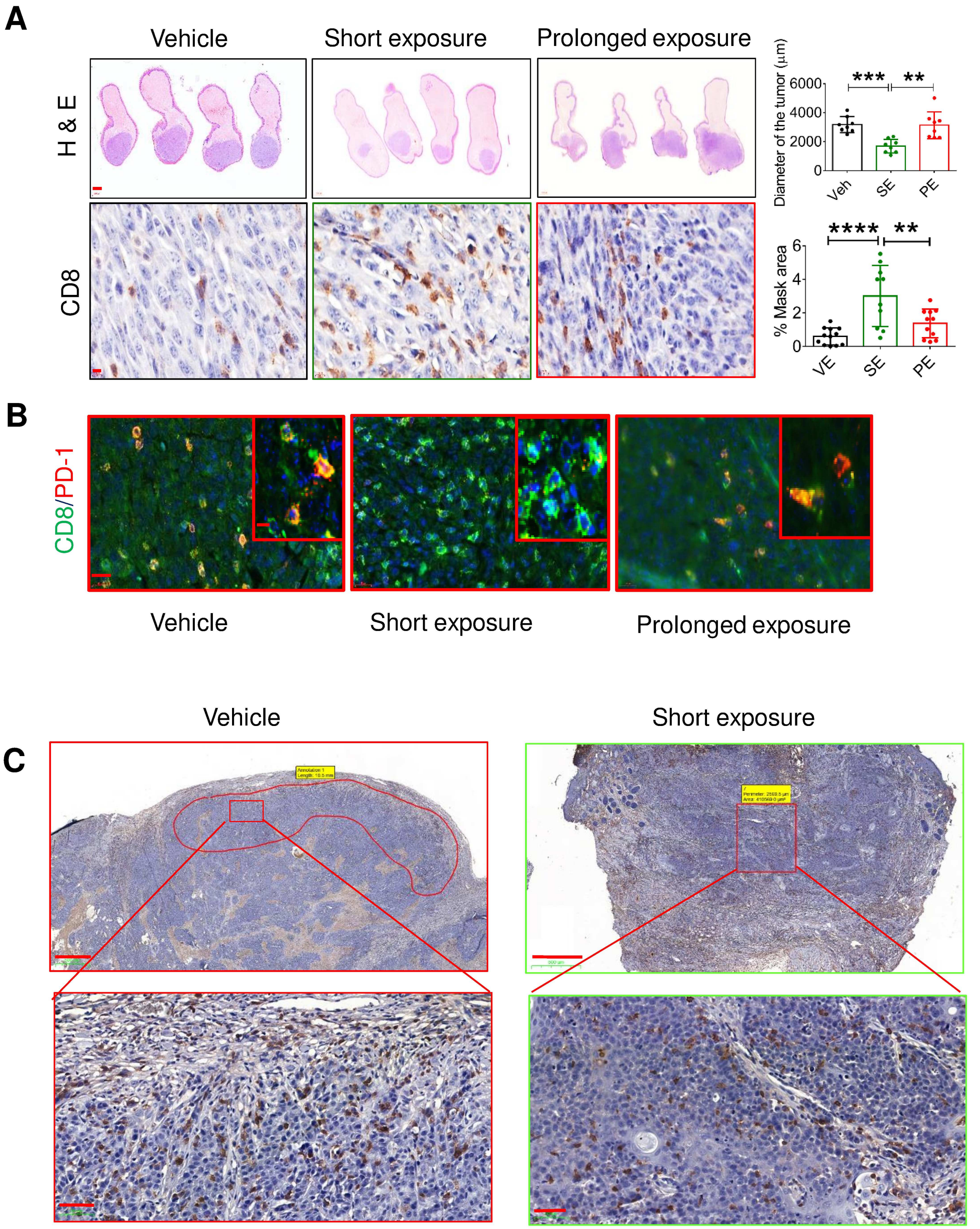
A
Vehicle
Short exposure
Prolonged exposure
H & E
CD8
Diameter of the tumor (μm)
6000
4000
2000
0
***
**
Veh
SE
PE
% Mask area
6
4
2
0
****
**
VE
SE
PE
B
CD8/PD-1
Vehicle
Short exposure
Prolonged exposure
Vehicle
Short exposure
C

Figure S4

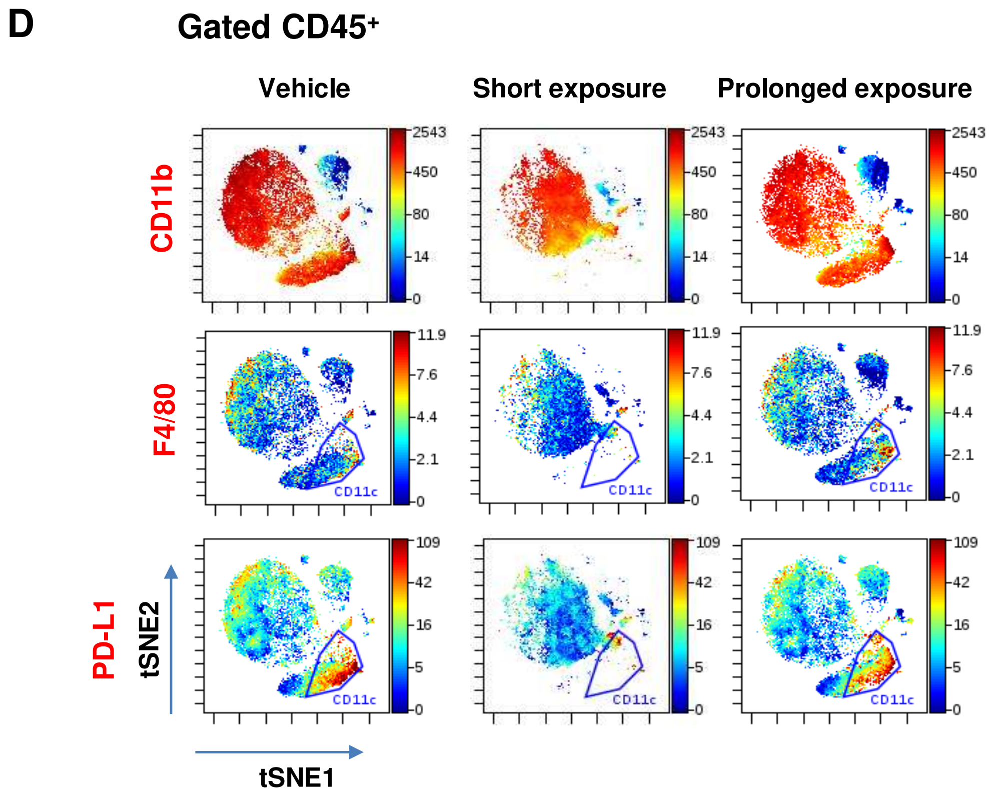
D
Gated CD45+
Vehicle
Short exposure
Prolonged exposure
CD11b
F4/80
PD-L1
tSNE2
tSNE1
CD11c
2543
450
80
14
0
11.9
7.6
4.4
2.1
109
42
16
5

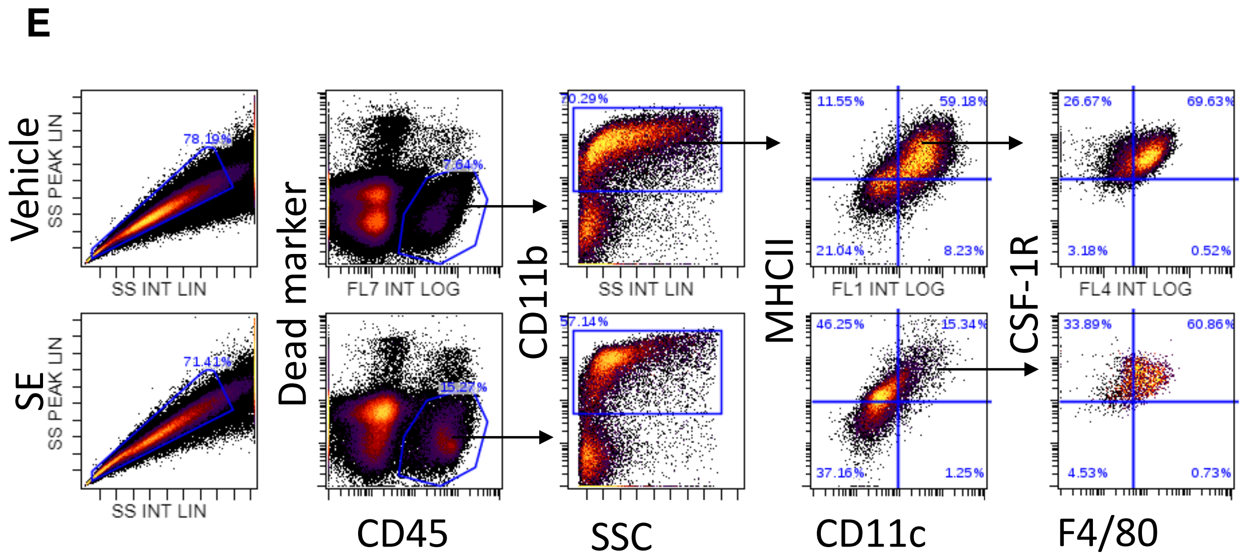
E
Vehicle
SE
SS PEAK LIN
SS INT LIN
78.19%
71.41%
Dead marker
FL7 INT LOG
7.64%
15.27%
CD45
CD11b
70.29%
57.14%
SSC
MHCII
11.55%
59.18%
21.04%
8.23%
46.25%
15.34%
37.16%
1.25%
FL1 INT LOG
CD11c
CSF-1R
26.67%
69.63%
3.18%
0.52%
33.89%
60.86%
4.53%
0.73%
FL4 INT LOG
F4/80

Figure S4

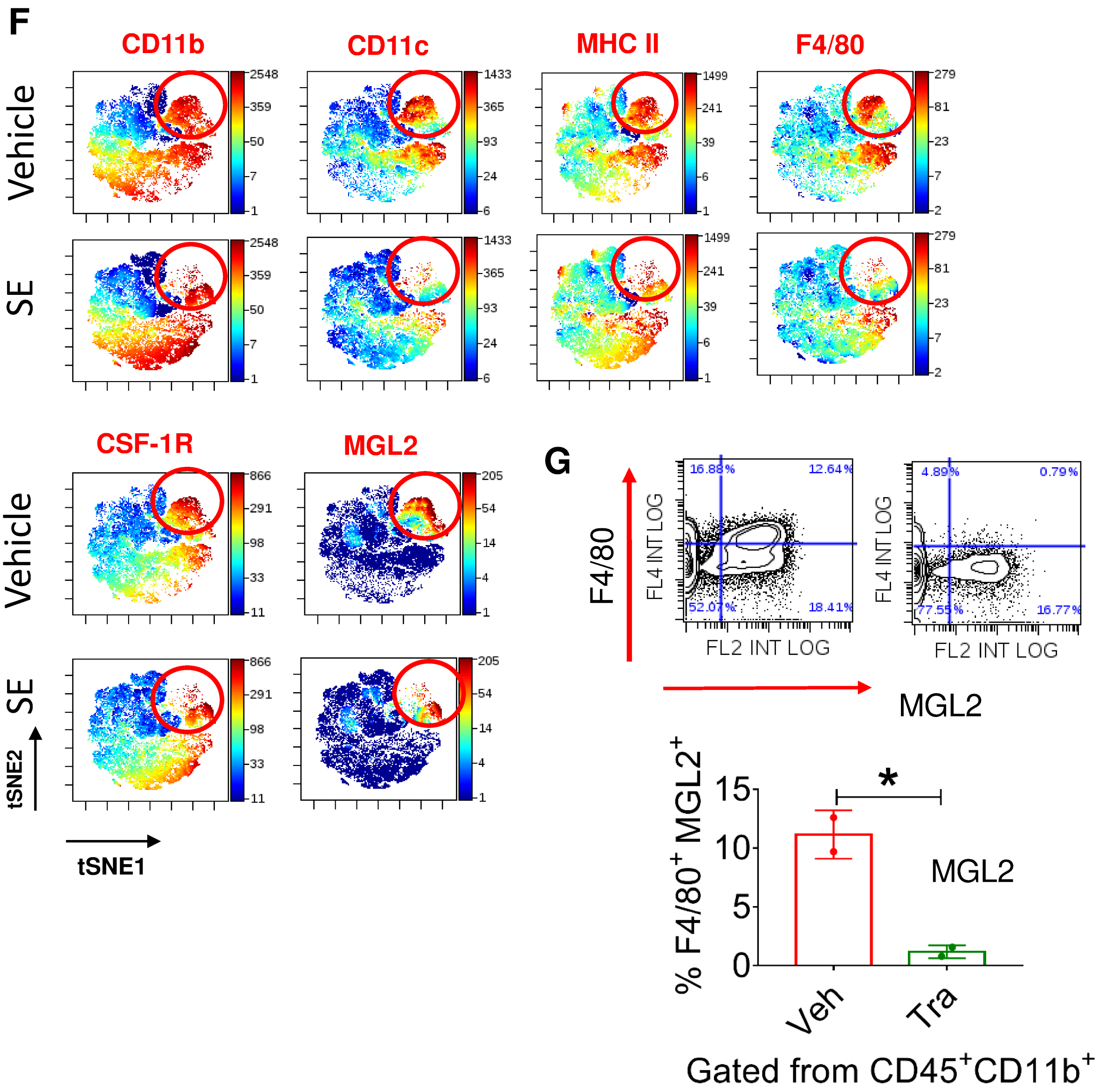
F
CD11b
CD11c
MHC II
F4/80
Vehicle
SE
CSF-1R
MGL2
tSNE2
tSNE1
G
F4/80
FL4 INT LOG
FL2 INT LOG
16.88%
12.64%
52.07%
18.41%
4.89%
0.79%
77.55%
16.77%
MGL2
% F4/80+ MGL2+
*
MGL2
Veh
Tra
Gated from CD45+CD11b+

Figure S4

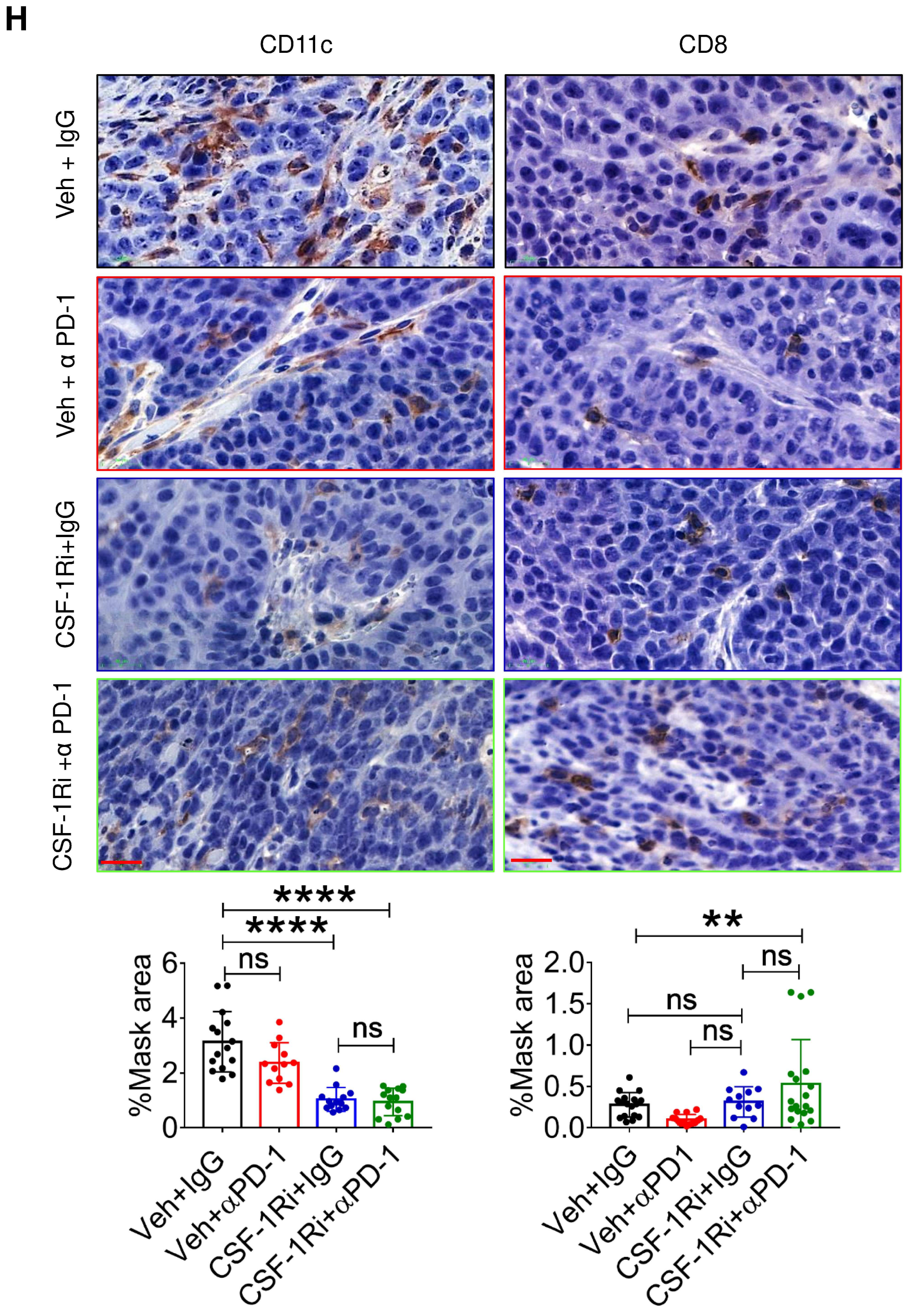
H
CD11c
CD8
Veh + IgG
Veh + α PD-1
CSF-1Ri+IgG
CSF-1Ri +α PD-1
****
****
ns
ns
%Mask area
0
2
4
6
Veh+IgG
Veh+αPD-1
CSF-1Ri+IgG
CSF-1Ri+αPD-1
**
ns
ns
ns
%Mask area
0.0
0.5
1.0
1.5
2.0
Veh+IgG
Veh+αPD1
CSF-1Ri+IgG
CSF-1Ri+αPD-1

Supplementary Figure S4. **(A)** Top – H&E stained images of tongues injected with 4NQO-T cells after exposure to vehicle, SE (7 days) trametinib or PE (25 days) trametinib (scale bars: 2000 μm). Bottom – IHC images and quantification of $CD8^+$ T cells in 4NQO-T tumors after treatment with trametinib for 7 and 25 days (scale bars: 20 μm). (**B**) IF co-staining (OPAL) of CD8 (green) and PD-1 (red) and merge (yellow) of 4NQO-T tumors treated as indicated (scale bars: 50 μm; inset 10 μm). (**C**) Pattern of infiltration of $CD8^+$ T cells in the vehicle-treated and SE trametinib-treated lip tissue samples (Scale bars: 500 μm; enlargements 50 μm). (**D**) viSNE plots of the CyTOF data showing CD11b, F4/80 and PD-L1 expression in $CD45^+$ cells from 4NQO-T tumors treated with vehicle, SE (5 days) trametinib, or PE (33 days) trametinib. (**E**) Flow cytometry analysis of $CD45^+$ cells based on their CD11b, CD11c, MHCII, CSF-1R and F4/80 expression. **(F)** viSNE plots representing flow data of the myeloid populations CSF-1R, CD11b, CD11c, MHCII, F4/80 and MGL2 ($CD301b^+$) shown for 4NQO-L tumors treated with vehicle or trametinib for 5 days (SE). (**G**) Flow cytometry dot plot analysis of MGL2 ($CD301b^+$) and F4/80 on $CD45^+CD11b^+$, when 4NQO-L tumors treated with a SE of trametinib or vehicle. (**H**) IHC analysis (top) and quantification (bottom) of CD11c and CD8 in tissues of 4NQO-L tumors treated with vehicle + IgG, vehicle + αPD-1, CSF-1Ri (CSF-1R inhibitor) + IgG, or CSF-1R + αPD-1 (scale bars: 20 μm). For statistics, an unpaired 2-sided *t*-test or one-way ANOVA was performed. $*p < 0.05$, $**p < 0.01$, $***p < 0.001$, and $****p < 0.0001$ were considered statistically significant. ns - not significant. Tra - trametinib, Veh - vehicle.

Figure S5

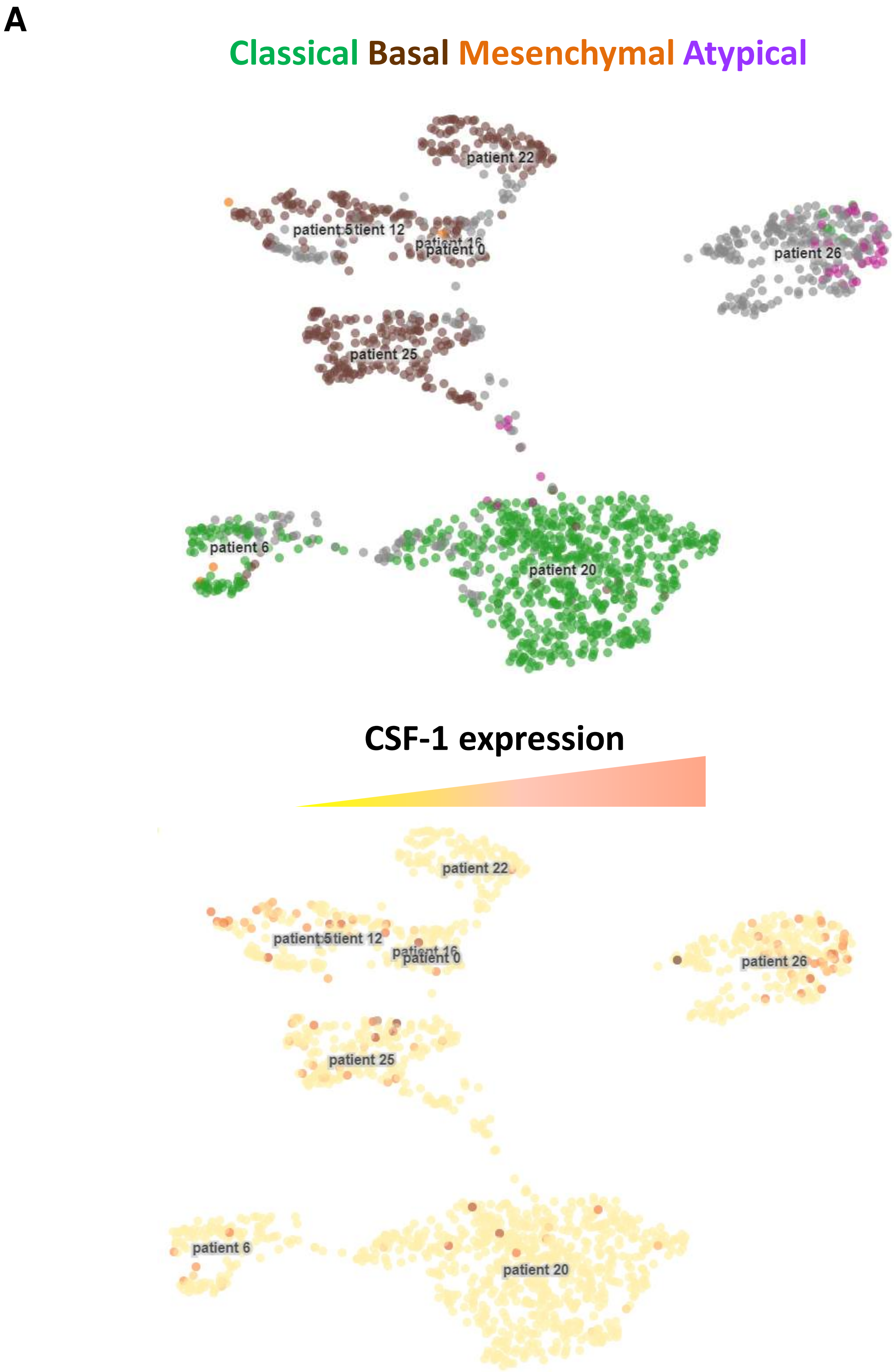
A
Classical Basal Mesenchymal Atypical
patient 22
patient 5
patient 12
patient 16
patient 0
patient 26
patient 25
patient 6
patient 20
CSF-1 expression
patient 22
patient 5
patient 12
patient 16
patient 0
patient 26
patient 25
patient 6
patient 20

Figure S5

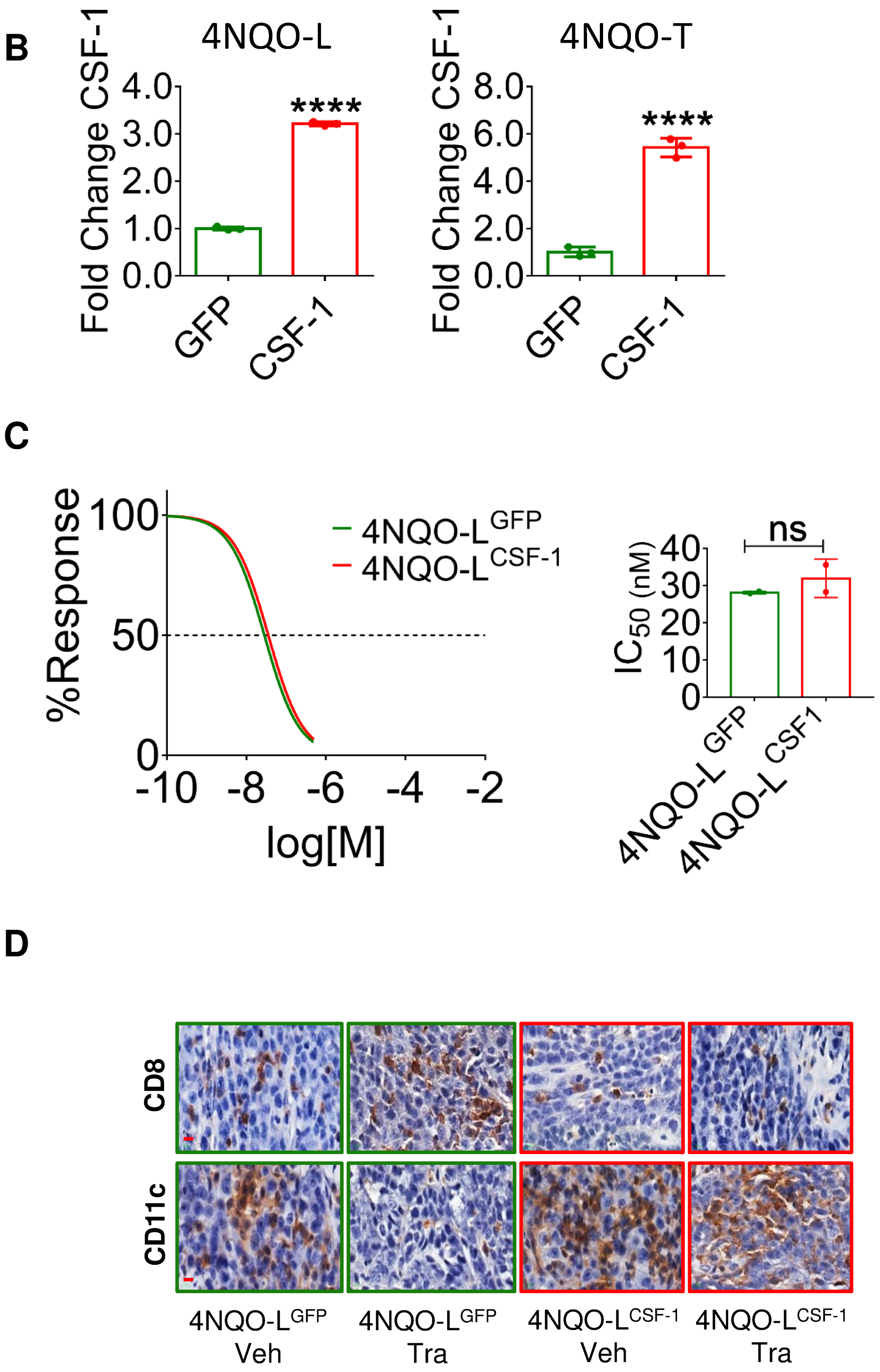
B
4NQO-L
4NQO-T
Fold Change CSF-1
4.0
3.0
2.0
1.0
0.0
8.0
6.0
4.0
2.0
0.0
****
GFP
CSF-1
C
%Response
100
50
0
-10
-8
-6
-4
-2
log[M]
4NQO-L^GFP
4NQO-L^CSF-1
IC50 (nM)
40
30
20
10
0
ns
4NQO-L GFP
4NQO-L CSF1
D
CD8
CD11c
4NQO-L^GFP Veh
4NQO-L^GFP Tra
4NQO-L^CSF-1 Veh
4NQO-L^CSF-1 Tra

Figure S5

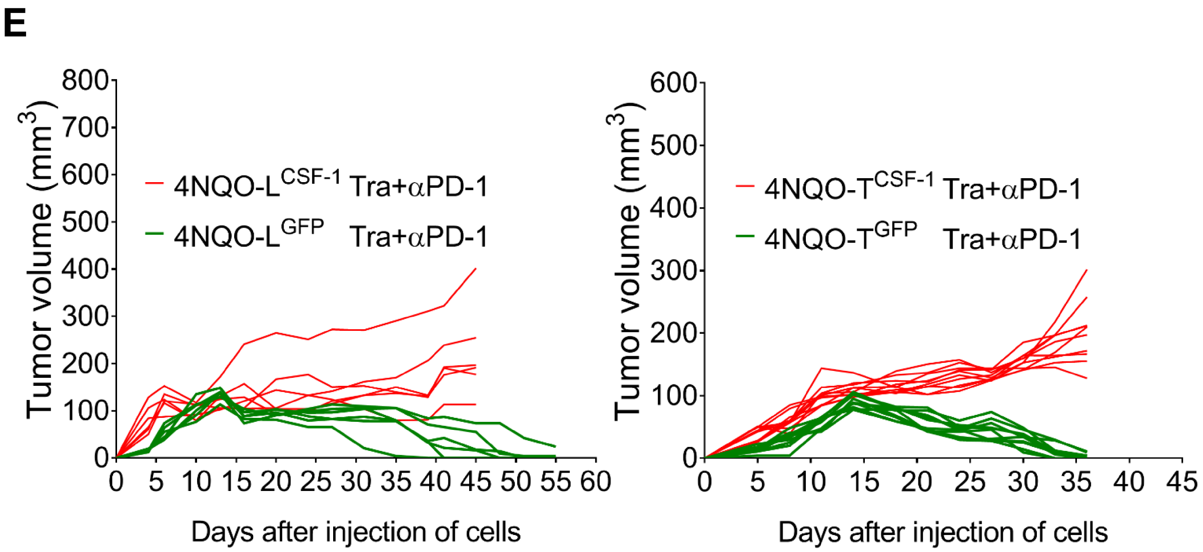
E
800
700
600
500
400
300
200
100
0
Tumor volume (mm3)
4NQO-LCSF-1 Tra+αPD-1
4NQO-LGFP Tra+αPD-1
0 5 10 15 20 25 30 35 40 45 50 55 60
Days after injection of cells
600
500
400
300
200
100
0
Tumor volume (mm3)
4NQO-TCSF-1 Tra+αPD-1
4NQO-TGFP Tra+αPD-1
0 5 10 15 20 25 30 35 40 45
Days after injection of cells

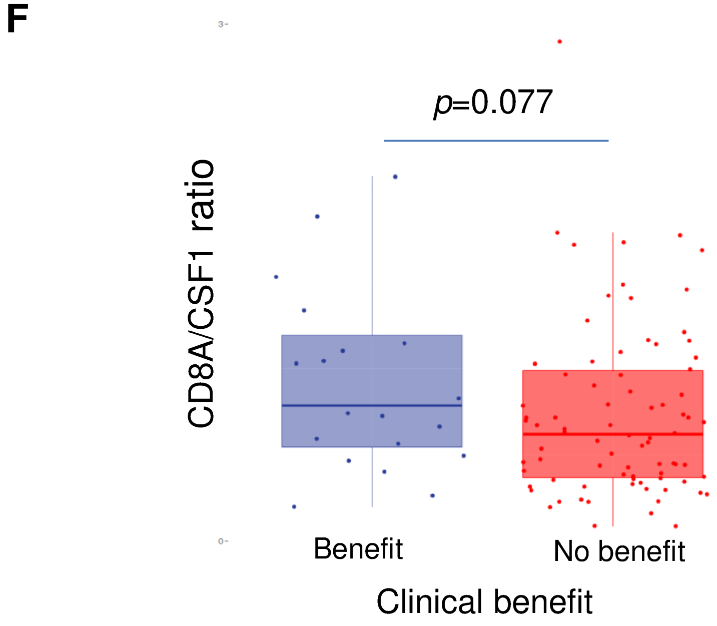
F
p=0.077
CD8A/CSF1 ratio
Benefit
No benefit
Clinical benefit

Figure S5

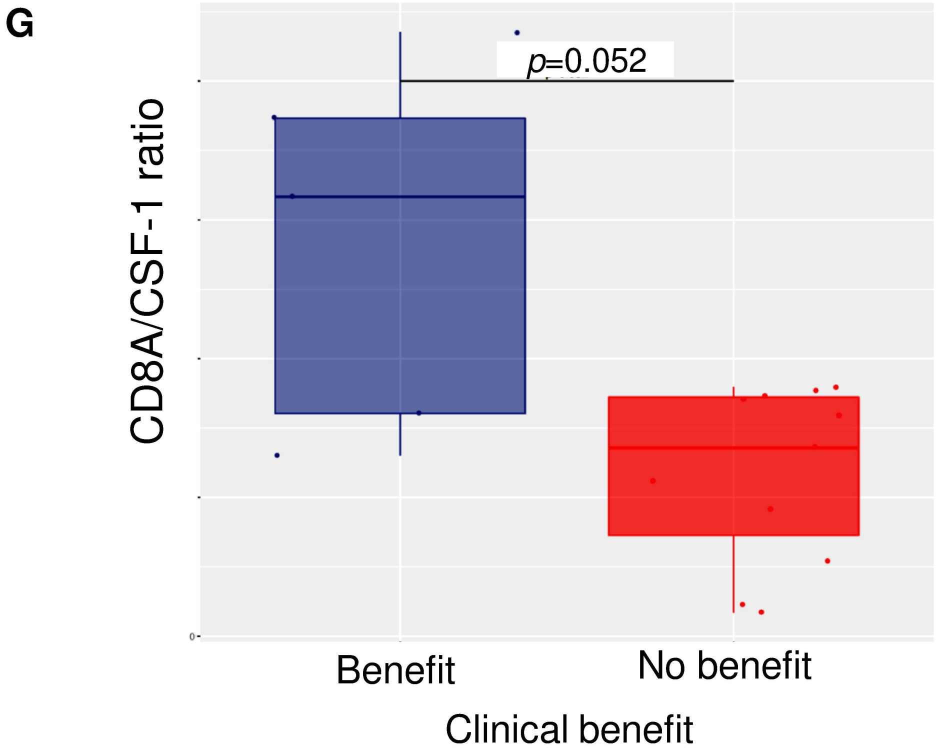
G
p=0.052
CD8A/CSF-1 ratio
0
Benefit
No benefit
Clinical benefit

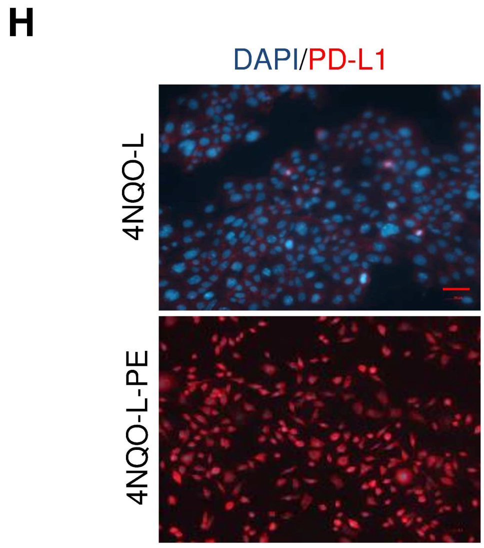
H
DAPI/PD-L1
4NQO-L
4NQO-L-PE

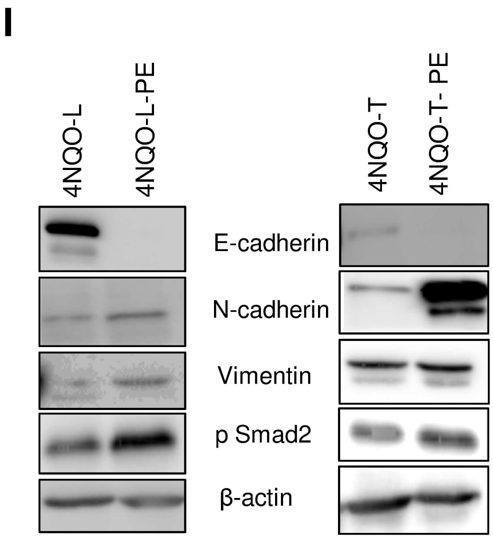
I
4NQO-L
4NQO-L-PE
4NQO-T
4NQO-T- PE
E-cadherin
N-cadherin
Vimentin
p Smad2
β-actin

Figure S5

J

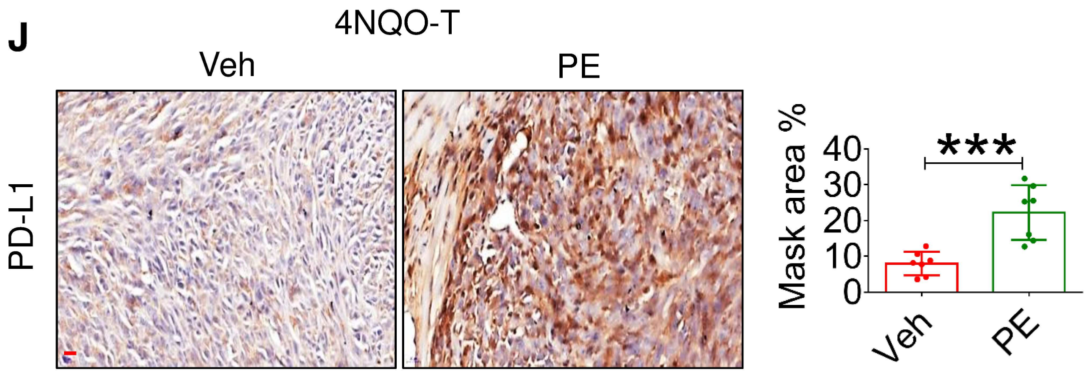
4NQO-T
Veh
PE
PD-L1
Mask area %
40
30
20
10
0
***
Veh
PE

K

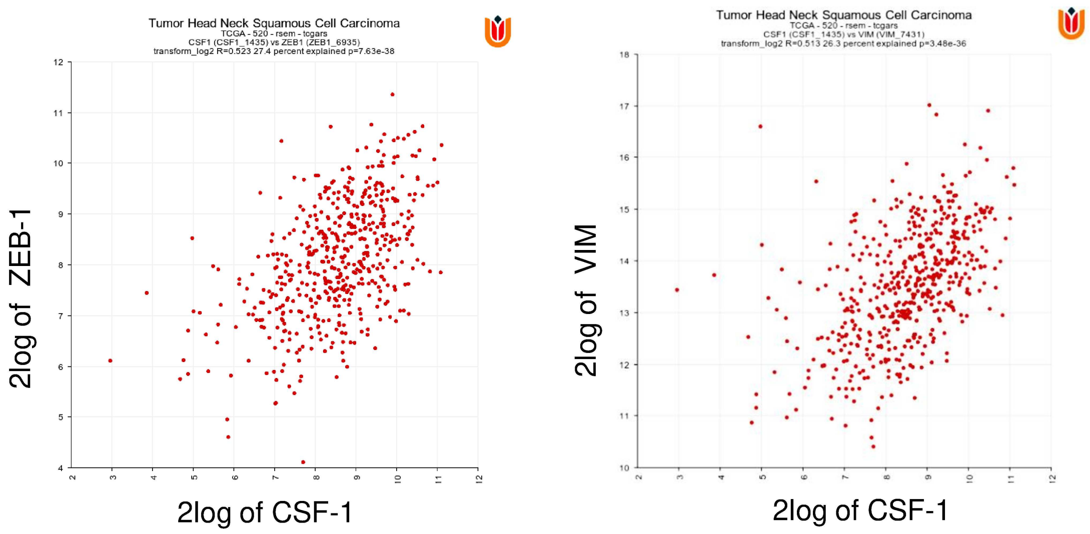
Tumor Head Neck Squamous Cell Carcinoma
TCGA - 520 - rsem - tcgars
CSF1 (CSF1_1435) vs ZEB1 (ZEB1_6935)
transform_log2 R=0.523 27.4 percent explained p=7.63e-38
2log of ZEB-1
2log of CSF-1
Tumor Head Neck Squamous Cell Carcinoma
TCGA - 520 - rsem - tcgars
CSF1 (CSF1_1435) vs VIM (VIM_7431)
transform_log2 R=0.513 26.3 percent explained p=3.48e-36
2log of VIM
2log of CSF-1

Figure S5

L

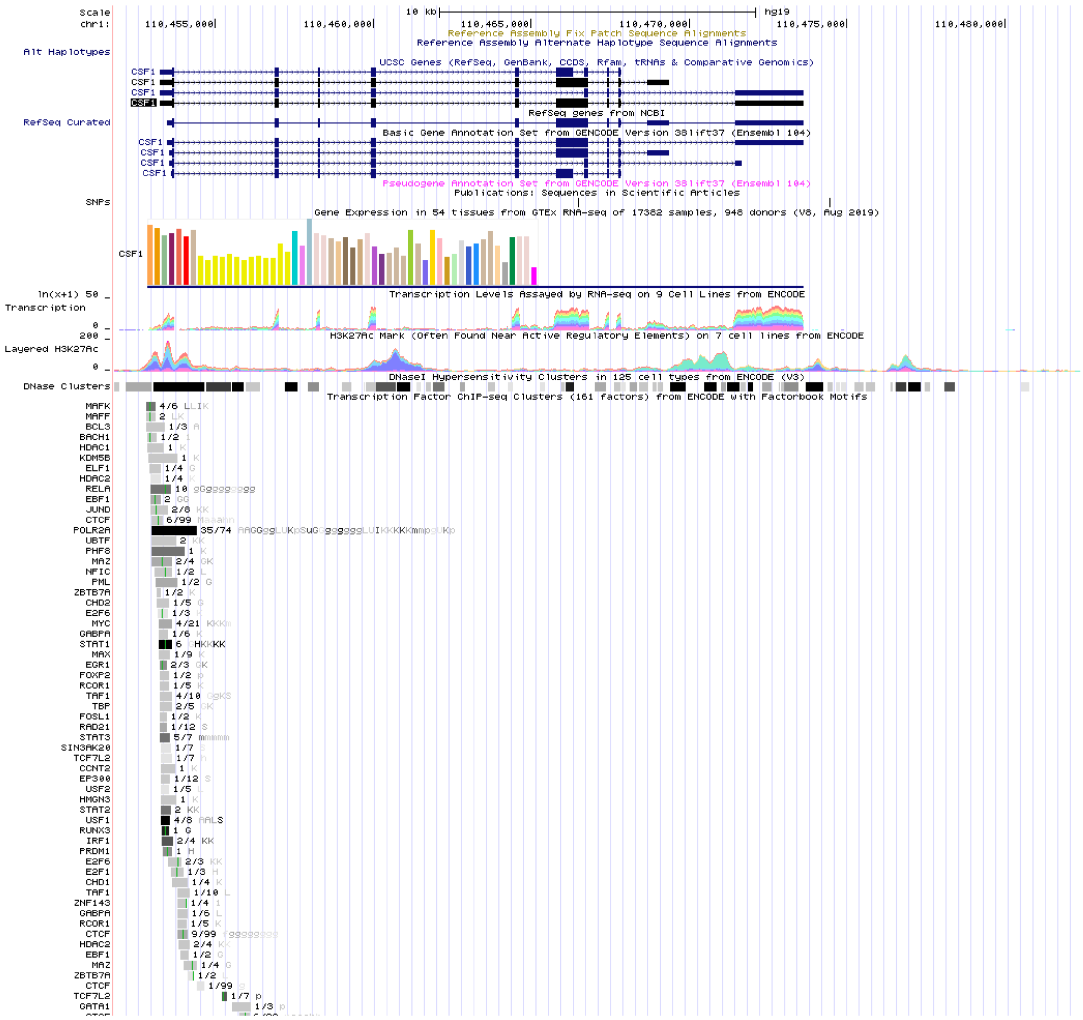
Scale
chr1:
10 kb
hg19
110,455,000
110,460,000
110,465,000
110,470,000
110,475,000
110,480,000
Reference Assembly Fix Patch Sequence Alignments
Reference Assembly Alternate Haplotype Sequence Alignments
Alt Haplotypes
UCSC Genes (RefSeq, GenBank, CCDS, Rfam, tRNAs & Comparative Genomics)
CSF1
RefSeq genes from NCBI
RefSeq Curated
Basic Gene Annotation Set from GENCODE Version 38lift37 (Ensembl 104)
Pseudogene Annotation Set from GENCODE Version 38lift37 (Ensembl 104)
Publications: Sequences in Scientific Articles
SNPs
Gene Expression in 54 tissues from GTEx RNA-seq of 17382 samples, 948 donors (V8, Aug 2019)
ln(x+1) 50
Transcription
Transcription Levels Assayed by RNA-seq on 9 Cell Lines from ENCODE
200
Layered H3K27Ac
H3K27Ac Mark (Often Found Near Active Regulatory Elements) on 7 cell lines from ENCODE
DNase Clusters
DNaseI Hypersensitivity Clusters in 125 cell types from ENCODE (V3)
Transcription Factor ChIP-seq Clusters (161 factors) from ENCODE with Factorbook Motifs
MAFK
MAFF
BCL3
BACH1
HDAC1
KDM5B
ELF1
HDAC2
RELA
EBF1
JUND
CTCF
POLR2A
UBTF
PHF8
MAZ
NFIC
PML
ZBTB7A
CHD2
E2F6
MYC
GABPA
STAT1
MAX
EGR1
FOXP2
RCOR1
TAF1
TBP
FOSL1
RAD21
STAT3
SIN3AK20
TCF7L2
CCNT2
EP300
USF2
HMGN3
STAT2
USF1
RUNX3
IRF1
PRDM1
E2F6
E2F1
CHD1
TAF1
ZNF143
GABPA
RCOR1
CTCF
HDAC2
EBF1
MAZ
ZBTB7A
CTCF
TCF7L2
GATA1

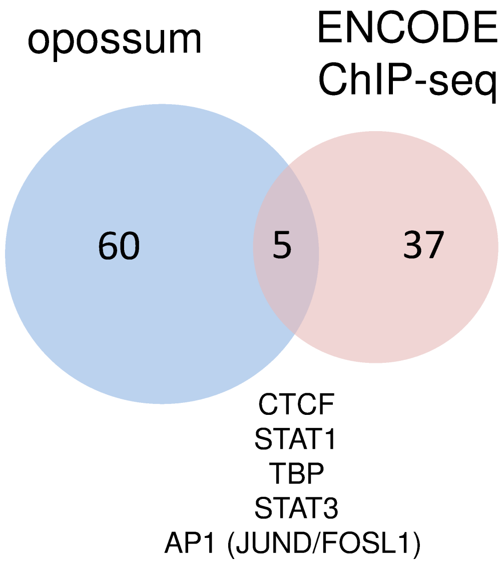
opossum
ENCODE ChIP-seq
60
5
37
CTCF
STAT1
TBP
STAT3
AP1 (JUND/FOSL1)

Figure S5

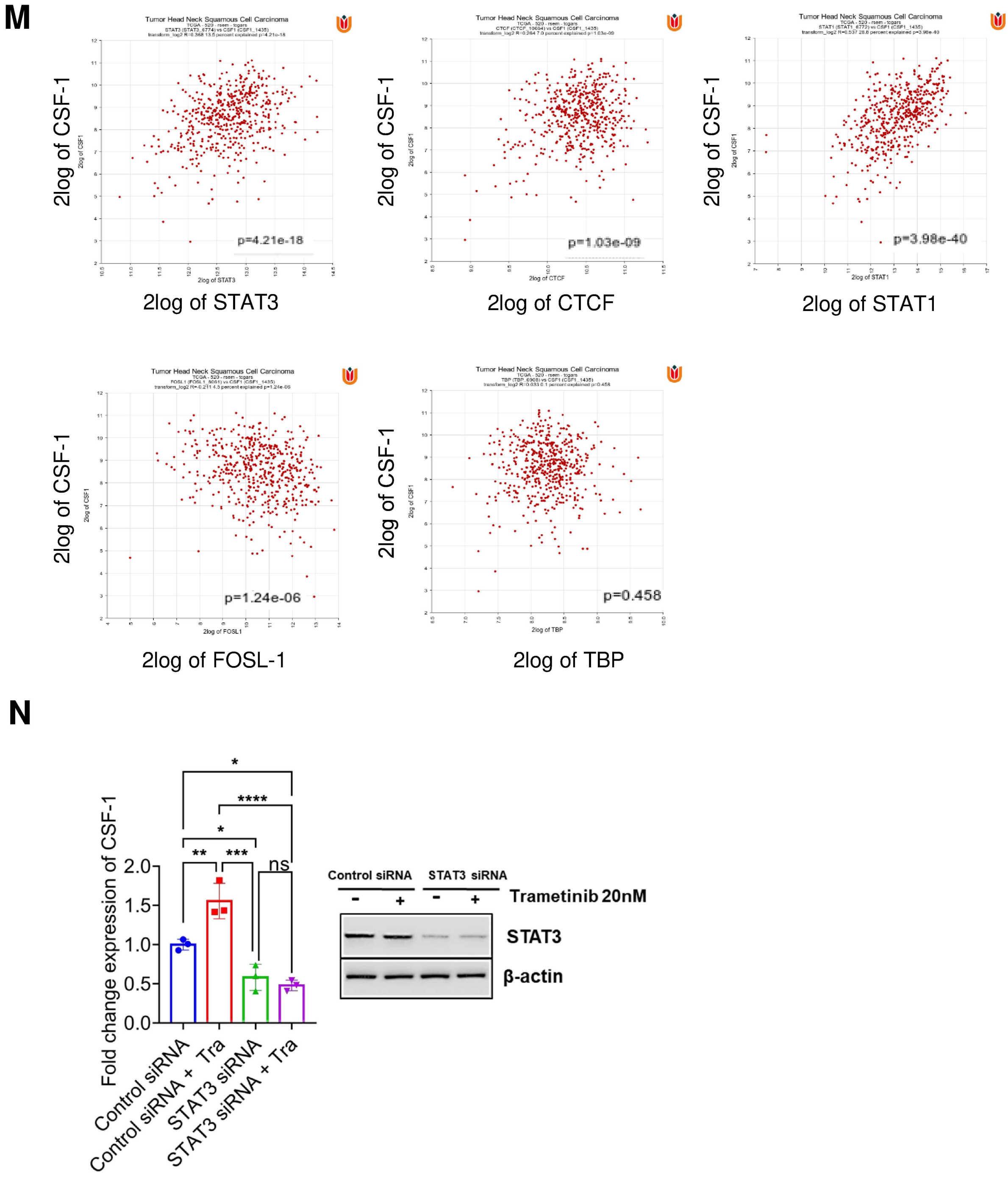
M
Tumor Head Neck Squamous Cell Carcinoma
2log of CSF-1
2log of STAT3
p=4.21e-18
2log of CTCF
p=1.03e-09
2log of STAT1
p=3.98e-40
2log of FOSL-1
p=1.24e-06
2log of TBP
p=0.458
N
Fold change expression of CSF-1
Control siRNA
Control siRNA + Tra
STAT3 siRNA
STAT3 siRNA + Tra
ns
Control siRNA
STAT3 siRNA
Trametinib 20nM
STAT3
β-actin

Supplementary Figure S5. **(A)** Single-cell analysis of CSF-1 expression in tumor cells of HNC patients. (**B**) mRNA levels of CSF-1 in 4NQO-L and 4NQO-T overexpressed with GFP or CSF-1. **(C)** Dose-response curve (left) of trametinib in 4NQO-L-GFP and 4NQO-L-CSF-1 cells, and IC_{50} values obtained in two independent experiments (right). (**D)** IHC staining of CD8 and CD11c in 4NQO-L^{GFP} and 4NQO-L^{CSF-1} tumors treated with trametinib for 5 days (SE) (scale bars: 50 µm). (**E)** Growth curves of single mice with 4NQO-L^{GFP} or 4NQO-L^{CSF-1} (left) and 4NQO-T^{GFP} or 4NQO-T^{CSF-1} (right) tumors treated with a combination of trametinib and αPD-1 (n = 6-10). (**F**) Box plots showing the relationship between the CD8A/CSF-1 ratio and the clinical benefits of αPD-1/PD-L1 treatment in 102 patients with head and neck squamous cell carcinoma (HNSCC) from the CLB-IHN cohort (based on RECIST criteria, clinical benefit was defined as complete response, partial response and stable disease of at least 6 months). A Mann-Whitney-Wilcoxon non-parametric test was used to compare the value of the CD8A/CSF-1 ratio between the two groups. **(G)** Box plots showing the value of the CD8A/CSF-1 ratio in a lung cancer cohort. (**H)** IF images showing the expression of PD-L1 in 4NQO-L and 4NQO-L-PE cells. (**I)** Western blot analysis showing the expression levels of E-cadherin, N-cadherin, vimentin, pSMAD2 and β-actin in 4NQO-L, 4NQO-L-PE, 4NQO-T and 4NQO-T-PE cells. (**J)** IHC images and quantification of PD-L1 expression in 4NQO-T tumors treated with vehicle and trametinib for 30 days (PE) (scale bar: 100 µm). **(K)** Correlation of expression of CSF-1 with ZEB1 and VIM, using the R2: Genomics Analysis and Visualization Platform. (**L**) ENCODE analysis of the promoter of CSF-1 and cross sectioning analysis of upregulated signature of TF in 4NQOs-PE cells and the TFs shown in ENCODE. (M) Correlation of expression of CSF-1 with STAT3, CTCF, STAT1, FOS-L and TBP, using the R2: Genomics Analysis and Visualization Platform. (N) Left- mRNA level expression of CSF-1 after silencing STAT3 in 4NQO-L PE cells treated with DMSO or trametinib (20nM). Right- STAT3 silencing was confirmed by western blot. For statistics, an unpaired 2-sided t-test or one-way ANOVA was performed. *$p < 0.05$; **$p < 0.01$; ***$p < 0.001$, ****$p < 0.0001$ were considered statistically significant. Tra - trametinib, Veh - vehicle.
